# Trans-Golgi network tethering factors regulate TBK1 trafficking and promote the STING-IFN-I pathway

**DOI:** 10.1038/s41421-024-00763-z

**Published:** 2025-03-18

**Authors:** Jinrui Wang, Shenghui Niu, Xiao Hu, Tianxing Li, Shengduo Liu, Yingfeng Tu, Zehua Shang, Lin Zhao, Pinglong Xu, Jingwen Lin, Lu Chen, Daniel D. Billadeau, Da Jia

**Affiliations:** 1 Key Laboratory of Birth Defects and Related Diseases of Women and Children, Department of Pediatrics, West China Second University Hospital, State Key Laboratory of Biotherapy, Sichuan University, Chengdu, Sichuan China; 2 MOE Laboratory of Biosystems Homeostasis and Protection and Innovation Center for Cell Signaling Network, Life Sciences Institute, Zhejiang University, Hangzhou, Zhejiang China; 3 Division of Oncology Research and Schulze Center for Novel Therapeutics, Mayo Clinic, Rochester, MN USA; 4 Development and Related Diseases of Women and Children Key Laboratory of Sichuan Province, West China Second University Hospital, Sichuan University, Chengdu, Sichuan China

**Keywords:** Membrane trafficking, Cell signalling

## Abstract

The cGAS-STING pathway mediates the innate immune response to cytosolic DNA, contributing to surveillance against microbial invasion or cellular damage. Once activated, STING recruits TBK1 at the trans-Golgi network (TGN), which in turn phosphorylates IRF3 to induce type I interferon (IFN-I) expression. In contrast to STING, little is known about how TBK1 is transported to the TGN for activation. Here, we show that multiple TGN tethering factors, a group of proteins involved in vesicle capturing, are indispensable for STING-IFN-I signaling. Deletion of TBC1D23, a recently reported tethering factor, in mice impairs the STING-IFN-I signaling, but with insignificant effect on STING-NF-κB signaling. Mechanistically, TBC1D23 interacts with TBK1 via the WASH complex subunit FAM21 and promotes its endosome-to-TGN translocation. Furthermore, multiple TGN tethering factors were reduced in aged mice and senescent fibroblasts. In summary, our study uncovers that TGN tethering factors are key regulators of the STING-IFN-I signaling and suggests that their reduction in senescence may produce aberrant STING signaling.

## Introduction

The cGMP-AMP (cGAMP) synthase (cGAS) senses cytosolic DNA and synthesizes the second messenger cGAMP, which activates the stimulator of interferon genes (STING) to trigger innate immune responses, leading to effective surveillance against microbial invasion or cellular damage^1,2^. Upon activation, STING recruits TANK-binding kinase 1 (TBK1) and activates interferon regulatory factor 3 (IRF3)^3^. TBK1-mediated phosphorylation of IRF3 triggers IRF3 dimerization and subsequent translocation to the nucleus, where IRF3 initiates the expression of transcription of type I interferons (IFN-I)^4^. This STING-IFN-I signaling is crucial for host defense and antitumor immunity. On the other hand, STING also mediates the activation of nuclear factor κB (NF-κB) via NEMO-IKKβ axis, which is critical for the full activation of TBK1^5^. Emerging evidence indicates that NF-κB can inhibit STING degradation to promote IFN-I induction^6^. Aberrant cGAS-STING signaling has been linked to cellular senescence and various human diseases, including infection, autoimmunity, tumor, and neurodegeneration^1,7–11^. For instance, the cGAS–STING pathway is known to drive aging-related inflammation in mice^12–14^. Cellular senescence often associates with increased DNA damage, either by the accumulation of cytoplasmic chromatin fragments or double-strand DNA breaks^14^. Interestingly, in both cases, mainly the STING-NF-κB signaling, but not the IFN-I signaling is more strongly activated in both cases^14,15^. Despite the importance, the mechanisms underlying different regulation of the IFN-I and NF-κB signalings downstream of STING remain poorly understood.

The cGAS–STING pathway involves multiple subcellular compartments and is dynamically modulated by vesicular trafficking^16,17^. STING, a transmembrane protein, resides in the endoplasmic reticulum (ER) at the resting state^18^. Upon binding to cGAMP, STING undergoes conformational change and COPII-mediated translocation from the ER to the cis-Golgi network (CGN) and further to the trans-Golgi network (TGN), where it assembles signalosomes with TBK1 and IRF3^17,18^. Subsequently, STING vesicles are recognized by AP-1 and delivered to lysosomes for degradation, terminating the signaling. Unlike STING, little is known about how TBK1 is translocated to the TGN^19–21^.

The TGN serves as a key platform for sorting and transporting proteins to various destinations, including endosomes, lysosomes, and the plasma membrane^22–27^. Golgins are a large family of tethering proteins at the Golgi, and selectively capture transport vesicles before SNARE-mediated fusion^28^. In mammalian cells, four golgins localize at the TGN (golgin-97, golgin-245, GCC88, GCC185)^29–31^. Among them, Golgin subfamily A member 1 (GOLGA1, also known as golgin-97) and Golgin subfamily A member 4 (GOLGA4, also known as golgin-245) anchor to the TGN through the binding of their GRIP domains to the small GTPase ADP-ribosylation factor-like protein 1 (Arl1)^32–36^. In the past years, it has become increasingly clear that golgin-97 and golgin-245 cooperate with the TBC1D23 and WDR11 complex to mediate endosome-to-TGN trafficking of various transmembrane proteins, including cation independent mannose-6-phosphate receptor (CI-MPR) and TGN46^37–43^. Hereafter, we refer to golgin-97, golgin-245, and related TBC1D23 and WDR11 complex as TGN tethering factors. While TGN tethering factors play a critical role in multiple cellular and physiological functions, their involvement in STING signaling is still unexplored.

In this study, we show that multiple TGN tethering factors are required for the activation of the STING-IFN-I signaling pathway. Deletion of TBC1D23 in mice predominantly impairs STING-IFN-I signaling and has limited effect on the STING-NF-κB signaling. Mechanistically, TBC1D23 promotes the endosome-to-TGN translocation of TBK1 but not STING. Furthermore, we observe a significant reduction in multiple TGN tethering factors in aged mice and senescent cells. These findings underscore the critical roles of TGN tethering factors in TBK1 translocation and STING-IFN-I signaling and suggest that their reduction could contribute to the different outputs of STING-IFN-I signaling and STING-NF-κB signaling during senescence.

## Results

### Oxidative stress promotes the reduction of multiple TGN tethering factors during senescence

**Fig. 1:**
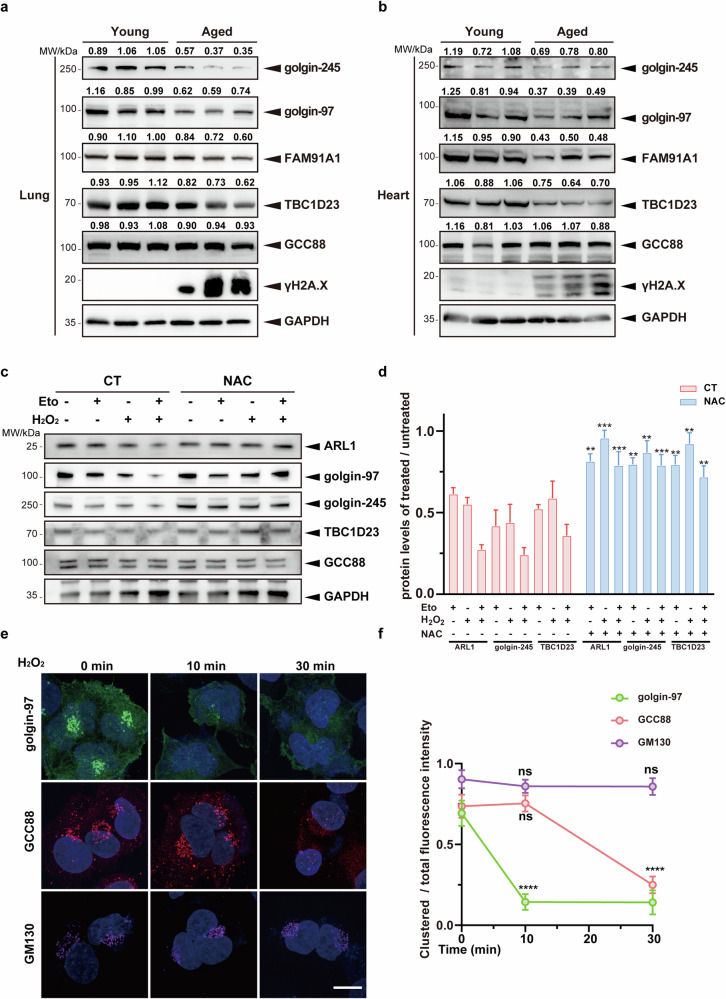
Multiple TGN tethering factors are reduced in aged mice or upon oxidative stress. **a**, **b** Lungs (**a**) or hearts (**b**) from young and aged mice were extracted and analyzed for expression levels of TGN tethering factors by immunoblotting. **c** Primary mouse lung fibroblasts were first treated with etoposide (50 μM) for 12 h or pretreated with H_2_O_2_ (200 μM) for 2 h and continued to be cultured in the presence or absence of etoposide (50 μM) for 12 h. N-acetylcysteine (NAC, 5 mM) was used to remove ROS. Untreated cells were used as a control, and the protein levels of TGN tethering factors were analyzed by immunoblotting. **d** Statistical analysis of golgin-245 and TBC1D23 expression levels, 30 cells were counted in each group. The levels were determined by normalizing the image gray values in **c**. Statistical results of golgin-97 and GCC88 are shown in Supplementary Fig. S1. **e** Immortalized human cerebral microvascular endothelial HCMEC/D3 cells were pretreated with H_2_O_2_ (500 μM). Distribution of Golgi proteins was analyzed by fluorescence confocal microscopy. Scale bar = 10 μm. **f** Quantifying the ratio of clustered protein fluorescence intensity to total fluorescence intensity within a cell (*n* = 30). One representative experiment of at least three independent experiments is shown. Data analyzed by two-tailed *t*-test and shown as mean ± SD (*n* ≥ 3). ns, not significant, *p* > 0.05; *p < 0.05; **p < 0.01; ***p < 0.001; ****p < 0.0001.

Senescence leads to multiple organelle stresses^44^. Altered structure and function of the Golgi apparatus have been reported in senescent cells^45^. However, the changes that TGN undergoes in aged mice are not well known. To investigate changes in TGN tethering factors during senescence, we analyzed their protein levels from multiple tissues (heart, liver, and lung) in young and aged mice (Fig. 1a, b; Supplementary Fig. S1a). Immunoblotting analysis revealed a significant decrease in the protein levels of multiple TGN tethering factors, including golgin-97, golgin-245, TBC1D23, and FAM91A1, across multiple tissues in aged mice. Interestingly, no significant reduction was observed for GCC88. Thus, many but not all, TGN tethering factors are reduced during aging.

Ireland et al. reported that H_2_O_2_ treatment in HeLa cells led to the degradation of Arl1, resulting in the degradation of golgin-97 and golgin-245^46^. To investigate whether oxidative stress during senescence induces the decrease of TGN tethering factors, we induced cellular senescence in primary mouse lung fibroblasts by treating them with etoposide and H_2_O_2_, individually or in combination. Senescent cells induced by either method exhibited a reduction in Arl1, golgin-97 and golgin-245, but not GCC88, relative to untreated cells (Fig. 1c, d; Supplementary Fig. S1b). Importantly, the reduction of TGN tethering factors could be rescued by the addition of antioxidant N-acetylcysteine (Fig. 1c, d; Supplementary Fig. S1b). Inhibition of proteasomal or lysosomal activities did not restore the reduction of TGN tethering factors, in agreement with studies by Ireland et al.^46^ (Supplementary Fig. S1c). In addition, the mRNA levels of TGN tethering factors did not change dramatically (Supplementary Fig. S1d). However, cycloheximide could partially rescue the reduction, suggesting that oxidative stress may reduce the level of TGN tethering factors by inhibiting their protein translation (Supplementary Fig. S1c)^47^. Together with studies from mouse tissues, these results suggest that oxidative stress leads to a reduction in multiple TGN tethering factors during senescence.

To further investigate how different TGN tethering factors respond to oxidative stress, we analyzed the changes of their cellular localization by confocal fluorescence imaging. Treatment of immortalized human cerebral microvascular endothelial HCMEC/D3 cells with 500 μM H_2_O_2_ for 10 min led to almost complete dispersion of the fluorescent GRIP domain from golgin-97 (EGFP-GRIP) (Supplementary Fig. S1e, f). Consistently, we also found that golgin-97 was already dispersed at 10 min. In contrast, GCC88 was not dispersed until the treatment time was extended to 30 min. Finally, GM130, a marker for the cis-Golgi network, did not disperse within our experimental time window (Fig. 1e, f). Together, these results indicate that Golgi proteins have different sensitivities to oxidative stress, which might explain why only certain TGN tethering factors are reduced during aging or upon oxidative stress.

### Multiple TGN tethering factors promote activation of the STING-IFN-I signaling pathway

**Fig. 2:**
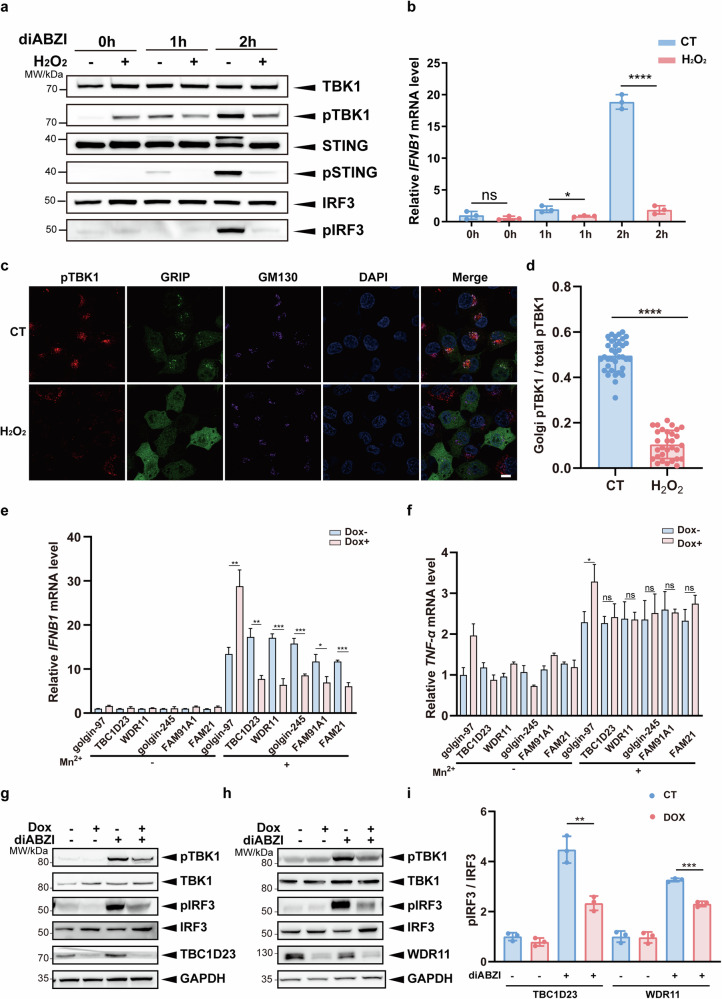
Multiple TGN tethering factors promote activation of the cGAS-STING-IFN-I signaling. **a** Immortalized human cerebral microvascular endothelial HCMEC/D3 cells were pretreated with H_2_O_2_ (200 μM) for 2 h. Endogenous STING activation was induced by diABZI (5 μM), and the phosphorylation levels of TBK1, STING and IRF3 were analyzed by immunoblotting at different time points. **b** Immortalized human cerebral microvascular endothelial HCMEC/D3 cells were pretreated with H_2_O_2_ (200 μM) for 2 h. The mRNA levels of *IFNB1* were analyzed by qPCR after diABZI (5 μM) treatment for various times. **c** HCMEC/D3 cells were processed as in **a**, and cells at the 1 h time point were analyzed by confocal fluorescence microscopy. Scale bar = 5 μm. **d** For images in **c**, Golgi-associated TBK1 phosphorylation intensity (using Golgi-resident protein GM130 as a reference) was counted and ratioed to the whole-cell signal intensity. **e**, **f** THP-1 cells stably expressing an inducible gene knockdown system were treated with dox (1 μM) for 72 h and followed by treatment with MnCl_2_ (200 μM) for 24 h. Cells were collected, and mRNA levels of *IFNB1* (**e**) and *TNF-α* (**f**) were determined by qPCR. **g**, **h** THP-1 cells stably expressing an inducible gene knockdown system were treated with dox (1 μM) for 72 h and followed by treatment with diABZI (5 μM) for 1 h. Phosphorylation of TBK1 and IRF3 was analyzed by immunoblotting. **i** Statistical analysis was performed after normalizing the image gray values of the experimental results in **g**, **h**. One representative experiment of at least three independent experiments is shown. Data are analyzed by two-tailed *t*-test and shown as mean ± SD (*n* ≥ 3). ns, not significant, *p* > 0.05; *p < 0.05; **p < 0.01; ***p < 0.001; ****p < 0.0001.

To investigate how oxidative stress regulates the STING signaling, we chose two different mouse models by administrating bromobenzene, which depletes glutathione levels and reduces body weights or alloxan, which generates reactive oxygen species^48,49^. The mice were then treated with STING agonists DMXAA. Surprisingly, the level of IFN-β, but not IL-6, was significantly reduced in both models (Supplementary Fig. S2a–d). To validate these results in cells, we first treated immortalized human cerebral microvascular endothelial HCMEC/D3 cells with or without H_2_O_2_, followed by treatment with the STING agonist diABZI, which induces a more dramatic activation of STING signaling^50^. TBK1, STING, and IRF3 phosphorylation, key events downstream of STING activation, were used to analyze STING activity. One hour after diABZI treatment, H_2_O_2_-pretreated cells showed slightly increased TBK1 phosphorylation at Ser172 (pTBK1) relative to control cells (Fig. 2a). However, at 2 h, the pTBK1, pSTING and pIRF3 strongly increased in control cells, but not in H_2_O_2_-pretreated cells (Fig. 2a). Consistently, H_2_O_2_ pre-treatment also significantly reduced the mRNA level of *IFNB1* relative to control cells at 2 h (Fig. 2b). To assess whether oxidative stress impaired TBK1 activation at the Golgi apparatus, we determined Golgi pTBK1 levels by confocal fluorescence microscopy. H_2_O_2_-pretreated cells displayed significantly lower pTBK1 levels at the Golgi apparatus (Fig. 2c, d). Thus, oxidative stress may inhibit the overall Golgi TBK1 signaling.

So far, our data indicate that oxidative stress leads to the degradation of TGN tethering factors and inhibits the STING signaling at the Golgi. To determine how TGN tethering factors regulate the STING signaling, we established doxycycline (dox)-inducible depletion of multiple tethering factors in THP-1 cells (Supplementary Fig. S3a), and treated the cells with Mn^2+^, an ion known to activate the STING signaling^51^. Depletion of all tethering factors, except for golgin-97 (see later discussion), dramatically suppressed the expression of *IFNB1* (Fig. 2e). Furthermore, knockdown of the WASH complex subunit FAM21, which functions in endosomal trafficking via direct interaction with TBC1D23, also inhibited *IFNB1* expression (Fig. 2e)^41^. Interestingly, depletion of these components, except for golgin-97, did not dramatically alter the expression of the inflammatory factor *TNF-α* (Fig. 2f). We further investigated the role of the TGN tethering factors in RIG-I-mediated IFN-I signaling by treating THP-1 cells with Poly I:C. Depletion of TGN tethering factors did not significantly affect *IFNB1* expression induced by Poly I:C (Supplementary Fig. S3b). Thus, TGN tethering factors are responsible for the activation of the STING-IFN-I signaling pathway.

To confirm the above results, we chose THP-1 cells depleted of TBC1D23 or WDR11 for further investigation. Depletion of TBC1D23 or WDR11 dramatically reduced pTBK1 levels upon diABZI treatment, relative to the control cells (Fig. 2g–i). Furthermore, the level of phosphorylated IRF3 was also decreased in both cell types. Altogether, these results indicate that multiple TGN tethering factors are indispensable for STING-IFN-I signaling.

### Deletion of *Tbc1d23* in mice predominantly impairs the STING-IFN-I signaling pathway

**Fig. 3:**
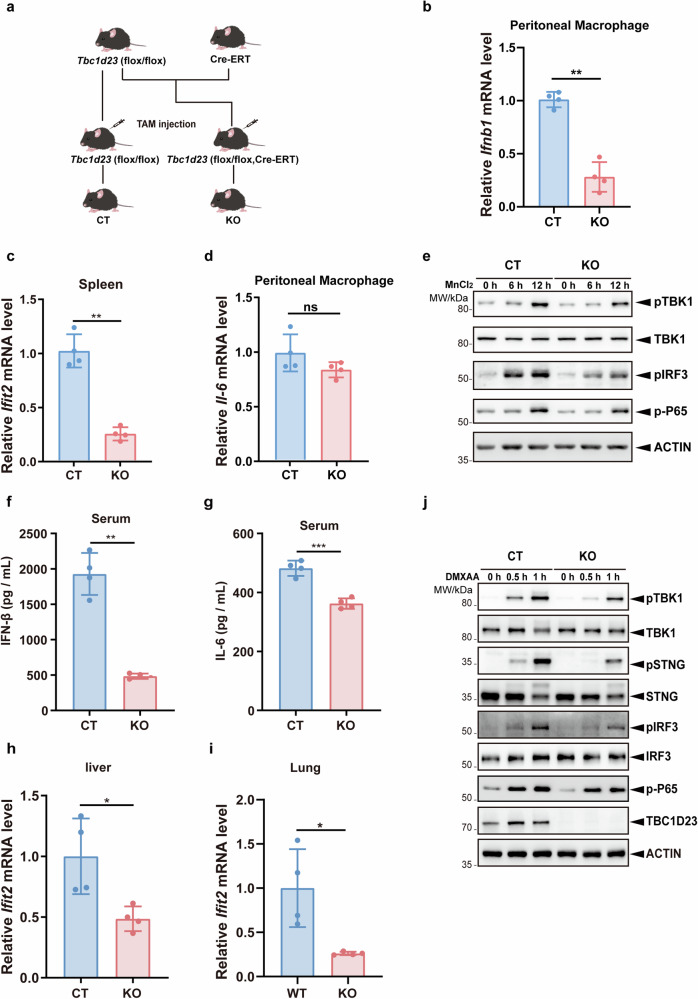
*Tbc1d23* knockout mice exhibit reduced STING-IFN-I signaling. **a** Generation of *Tbc1d23* conditional knockout mouse. **b** CT or *Tbc1d23* KO mice were injected intraperitoneally with 40 mg/kg of MnJβ adjuvant (1.4 mg/mL) dissolved in saline. Ten hours later, peritoneal macrophages were collected, and the expression levels of *Ifnb*1 were analyzed by qPCR. **c** CT or *Tbc1d23* KO Mice were injected intraperitoneally with 40 mg/kg of MnJβ adjuvant (1.4 mg/mL) dissolved in saline. Ten hours later, Spleens were collected, and the expression levels of *Ifit2* were analyzed by qPCR. **d** CT or *Tbc1d23* KO Mice were injected intraperitoneally with 40 mg/kg of MnJβ adjuvant (1.4 mg/mL) dissolved in saline. Ten hours later, peritoneal macrophages were collected, and the expression levels of *Il-6* were analyzed by qPCR. **e** BMDM cells obtained from CT or *Tbc1d23* KO mice were treated with MnCl_2_ (200 μM) various times, and then subjected to immunoblotting analysis. **f**–**i** CT or *Tbc1d23* KO Mice were injected intraperitoneally with 25 mg/kg of DMXAA (5 mg/mL) dissolved in 7.5% NaHCO_3_. Three hours later, the levels of serum IFN-β (**f**) and IL-6 (**g**) were determined with an ELISA kit. Organs from each mouse were extracted, and the expression levels of *Ifit2* were determined by qPCR (**h,** **i**). **j** BMDM cells obtained from CT or *Tbc1d23* KO mice were treated with DMXAA (25 μg/mL) for various time points, and then subjected to immunoblotting analysis. One representative experiment of at least three independent experiments is shown. Data analyzed by two-tailed *t*-test and shown as mean ± SD (*n* ≥ 3). ns, not significant, *p* > 0.05; *p < 0.05; **p < 0.01; ***p < 0.001; ****p < 0.0001.

As TBC1D23 functions as a bridge protein between golgin-97/245 and the WDR11 complex, we next focused on TBC1D23 and investigated how TBC1D23 regulates the STING-IFN-I signaling in vivo^38,41,42^. We first generated *Tbc1d23* floxed mice, which harbored one flox site at the intron between exons 2 and 3 and another flox site at the intron between exons 3 and 4 (Supplementary Fig. S4a). Exons 3 could be deleted via Cre-mediated recombination to introduce a frameshift (Supplementary Fig. S4a). To obtain *Tbc1d23* ^–/–^ (KO) mice, *Tbc1d23* (flox/flox) mice were first crossed with Cre-ERT mice to obtain *Tbc1d23* (flox/flox, Cre-ERT), and 4-week-old mice were then administered with tamoxifen (TAM) via intraperitoneal injection (Fig. 3a; Supplementary Fig. S4b). Deletion of *Tbc1d23* was confirmed by quantitative RT-PCR in the liver and lung (Supplementary Fig. S4c, d).

To assess the function of TBC1D23 in vivo, *Tbc1d23* KO or control (CT) mice were injected intraperitoneally with MnJβ adjuvant, a colloidal manganese salt, and the expression of multiple STING-regulated genes was analyzed^52^. Deletion of *Tbc1d23* significantly decreased *Ifnb1* mRNA levels in peritoneal macrophages and that of *Ifit2* (IFN-induced protein with tetratricopeptide repeats 2) in the spleen (Fig. 3b, c). In contrast, no significant difference in *Il-6, Il-1β, Tnf-α* expression was detected (Fig. 3d; Supplementary Fig. S4e, f). Next, we isolated bone marrow derived macrophages (BMDMs) from CT or *Tbc1d23* KO mice and treated the cells with Mn^2+^. Similar to the mouse study, deletion of *Tbc1d23* in BMDMs significantly reduced the expression of *Ifnb1*, which was 20% of that in CT mice (Supplementary Fig. S4g). Conversely, deletion of *Tbc1d23* only mildly decreased the mRNA level of *Il-6* (80% of that in CT mice) (Supplementary Fig. S4h). Moreover, deletion of *Tbc1d23* significantly impaired phosphorylation of TBK1, STING, and IRF3, but did not dramatically affect the levels of phosphorylated p65 (Fig. 3e). Together, these results indicate that deletion of TBC1D23 predominantly impairs Mn^2+^-induced STING-IFN-I signaling.

In addition to Mn^2+^, we also examined the effect of the STING agonist DMXAA on *Tbc1d23* KO or CT mice^53^. Similar to Mn^2+^, DMXAA administration dramatically reduced the IFN-β protein level in the serum of *Tbc1d23* KO mice relative to CT mice (Fig. 3f). On the other hand, the IL-6 protein level was only slightly reduced (Fig. 3g). Furthermore, the mRNA levels of *Ifit2* were significantly downregulated in both the livers and lungs of *Tbc1d23* KO mice compared to CT mice (Fig. 3h, i). When BMDMs isolated from *Tbc1d23* KO or CT mice were treated with DMXAA, the mRNA level of *Ifnb1* was significantly reduced in *Tbc1d23* KO mice (Supplementary Fig. S4i). Conversely, the mRNA levels of *Il-6* and *Tnf-α* were unchanged or slightly decreased (Supplementary Fig. S4j, k). Similar to Mn^2+^, DMXAA stimulation led to significantly reduced phosphorylation of TBK1, STING, and IRF3 in *Tbc1d23* KO mice (Fig. 3j). These results indicate that deletion of TBC1D23 strongly inhibits the induction of type I interferon by STING in mice but has no significant effect on the expression of inflammatory factors.

### TBC1D23 promotes the translocation and activation of TBK1

TBK1 is a soluble protein widely distributed in cells; however, it could be recruited to various organelles (e.g., endosomes and mitochondria) to drive multiple biological processes under different circumstances^54–57^. However, it is unknown how TBK1 is transported to the TGN, where it assembles with TBK1 and IRF3. To investigate the distribution of TBK1 on vesicles, we co-transfected COS7 cells with EGFP-TBK1 and various RFP-tagged vesicle markers and performed live-cell imaging. RFP-FYVE, RFP-CD63, and RFP-LAMP1 were used to label early endosomes, late endosomes, and lysosomes, respectively^58^. TBK1 formed puncta on the surface of FYVE- or CD63-positive structures; in contrast, TBK1 was mostly found to be internalized in LAMP1-positive structures (Supplementary Fig. S5a). Furthermore, TBK1 puncta moved along FYVE-positive structures (Supplementary Fig. S5a and Video S1). We and others have previously shown that the WASH complex is localized on endosomal vesicles, and a direct interaction between the WASH complex subunit FAM21 and TBC1D23 is critical for endosome-to-TGN transport of certain cargoes^39,41^. Thus, we investigated the subcellular distribution of TBK1 and FAM21 by fluorescence confocal microscopy. Both endogenous or exogenously expressed TBK1 displayed strong co-localization with FAM21 and RFP-FYVE (Supplementary Fig. S6a, b). Live-cell imaging of COS7 cells co-transfected with EGFP-TBK1, TBC1D23-mCherry, and BFP-FYVE also revealed that TBK1 and TBC1D23 co-localized on FYVE-positive structures (Supplementary Video S2). Thus, TGN tethering factors, such as TBC1D23, may regulate translocation of endosomal TBK1 to the TGN via FAM21.

**Fig. 4:**
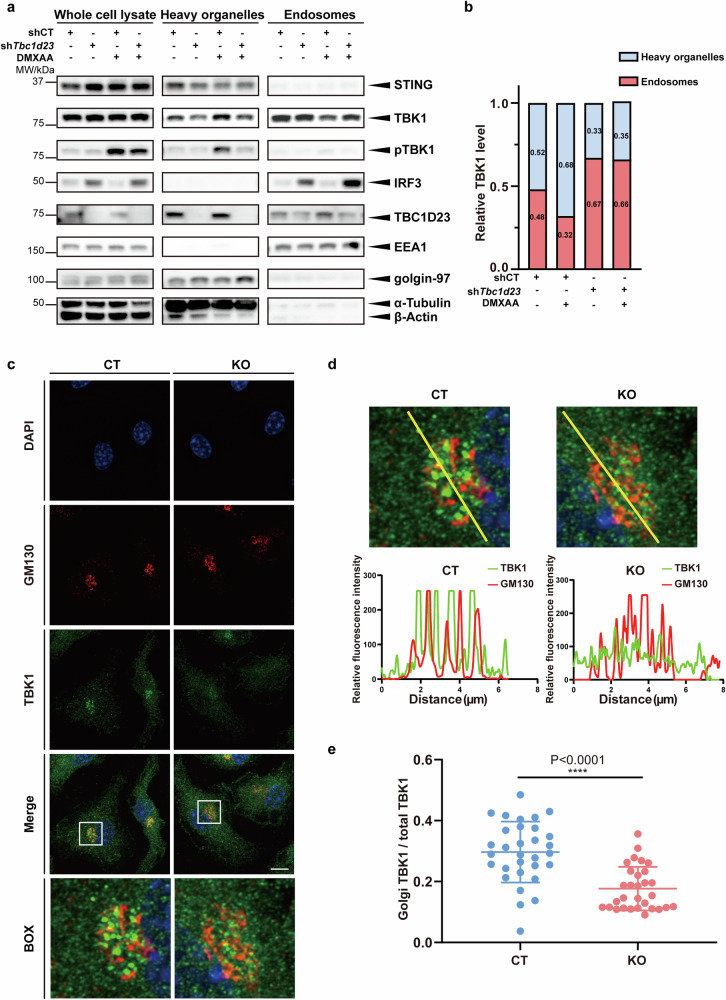
TBC1D23 promotes TBK1 translocation to the Golgi apparatus. **a** MEFs stably expressing TBC1D23-targeting shRNA (sh*Tbc1d23*), or control shRNA (shCT) were treated with DMXAA (25 μg/mL) for 30 min. Endosomes and heavy organelles (Golgi, endoplasmic reticulum, mitochondria, etc.) were isolated, and subjected to immunoblotting. EEA1 and golgin-97 were used to indicate endosomes and Golgi, respectively. **b** Quantification based on grey values. Results from three different experiments in (**a**) were combined, and data were averaged. **c**, **d** BMDM cells were treated with DMXAA (25 μg/mL) for 30 min. Golgi-association of TBK1 was analyzed by confocal fluorescence microscopy, using Golgi-resident protein GM130 as a reference. Line scans show the related intensity profiles of TBK1 (green) and GM130 (red). Scale bar = 5 μm. **e** Quantitative analysis of the ratio of Golgi TBK1 to total TBK1, as shown in (**c**). One representative experiment of at least three independent experiments is shown. Data are analyzed by two-tailed *t*-test and shown as mean ± SD (*n* ≥ 3). ns, not significant, *p* > 0.05; *p < 0.05; **p < 0.01; ***p < 0.001; ****p < 0.0001.

To understand how TBC1D23 regulates the intracellular trafficking of TBK1 upon STING activation, we knocked down *Tbc1d23* with shRNA in MEFs. We then performed subcellular fractionation in DMXAA-treated and non-treated cells, along with control cells. TBC1D23 could be found in both endosomal and heavy organelle fractions, which contained the Golgi apparatus, endoplasmic reticulum, mitochondria, and others (Fig. 4a). In contrast, IRF3 was predominantly found in endosomal fractions (Fig. 4a). DMXAA strongly activated the STING signaling, as evidenced by the increase of total pTBK1 (Fig. 4a). Consistent with previous studies, pTBK1 was mainly found in heavy organelles (Fig. 4a). When STING was activated, TBK1 in endosomal fractions was significantly decreased, accompanied by an increase in heavy organelle fractions, indicating that TBK1 translocated from endosomes to heavy organelles (Fig. 4a, b). Using protein proximity labeling, we also found that diABZI treatment decreased the association between TBK1 and endosomal proteins, such as RAB7A (Supplementary Fig. S7a–c); in the meantime, the association of TBK1 with TGN proteins, such as golgin97, increased (Supplementary Fig. S7a–c). Depletion of *Tbc1d23* in MEFs drastically decreased the level of pTBK1, consistent with our previous results (Fig. 4a, b). Depletion of *Tbc1d23* also hindered the translocation of TBK1 from endosomes to heavy organelles (Fig. 4a, b). These results suggest that STING activation leads to the translocation of TBK1 from endosomes to heavy organelles, and TBC1D23 is indispensable for the process.

Immunofluorescence analysis of BMDMs isolated from CT mice revealed that TBK1 was significantly enriched at the Golgi apparatus upon DMXAA treatment (Fig. 4c–e). Deletion of TBC1D23 significantly decreased TBK1 localization at the Golgi (Fig. 4c–e). Similarly, depletion of TBC1D23 also inhibited TBK1 activation at the Golgi in MEFs (Supplementary Fig. S8a–c).

TBK1 is a cytoplasmic protein but can be recruited to multiple subcellular organelles through binding to adapter proteins such as TANK, OPTN, TBKBP1, and NAP1^59,60^. TBK1, via its C-terminal domain, interacts with these adapters, and utilizes its N-terminal domain to contact STING (Supplementary Fig. S9a)^59–62^. L693 of TBK1 is critically involved in the binding with the adapter proteins^59,60^. Indeed, TBK1 L693Q significantly reduced the association with membrane, as determined by immunofluorescence or cellular fractionation (Supplementary Fig. S9b–d). To assess whether endosomal localization of TBK1 is important for the STING signaling, we generated *Tbk1/Ikkε* double knockout B16 cells and rescued with TBK1 WT or L693Q. In comparison with TBK1 WT, L693Q significantly reduced the phosphorylation levels of TBK1 (p-TBK1) and STING (p-STING) upon STING agonist treatment (Supplementary Fig. S9e, f). Activated STING led to eventual TBK1 degradation. However, TBK1 degradation was compromised in cells expressing L693Q (Supplementary Fig. S9e). To further confirm the endosomal localization of TBK1 in regulating the STING signaling, we designed a rapamycin-induced endosome-targeting method based on the FKBP-FRB system (Supplementary Fig. S10a)^63–65^. In the absence of DMXAA, rapamycin addition induced the phosphorylation of TBK1, but not that of STING; In the presence of DMXAA, rapamycin addition significantly increased the phosphorylation levels of both TBK1 and STING (Supplementary Fig. S10b, c). Taken together, our results suggest that TBC1D23 promotes endosome-to-Golgi translocation and activation of TBK1.

### TBC1D23 interacts with TBK1 via FAM21 and regulates the STING-IFN-I signaling pathway

**Fig. 5:**
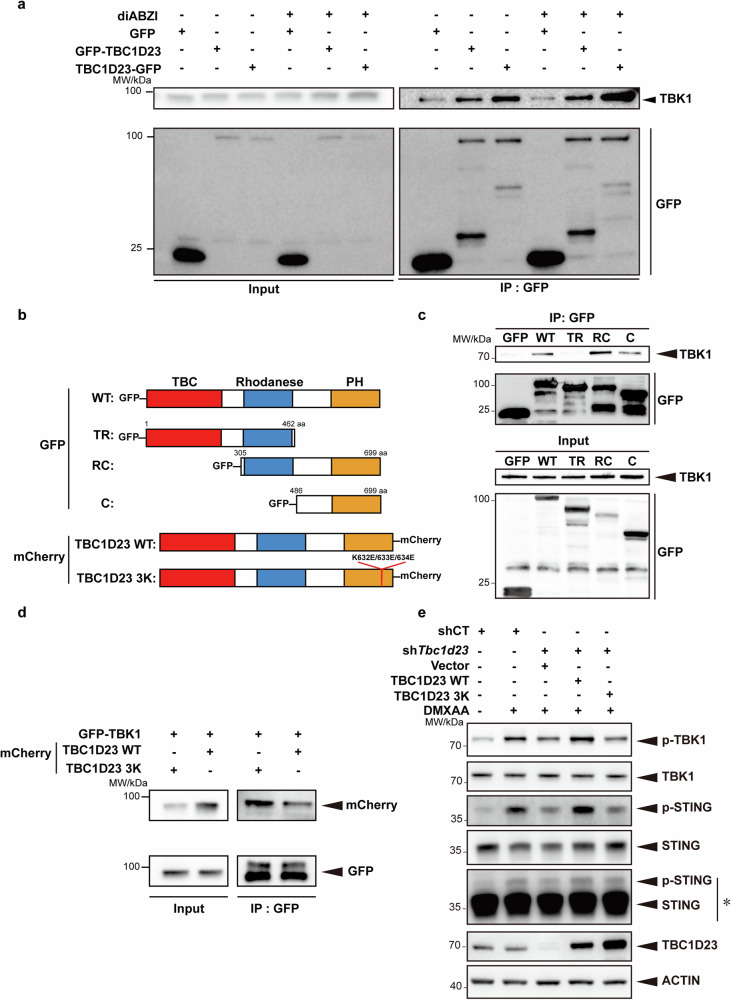
TBC1D23 interacts with TBK1 and regulates the STING-IFN-I signaling. **a** THP-1 cells stably expressing GFP, GFP-TBC1D23, or TBC1D23-GFP were treated with or without diABZI and subjected to immunoprecipitation using GFP beads. Bound TBK1 was analyzed by immunoblotting. **b** Schematic diagram of full-length and various truncations of TBC1D23 used in the experiment. Red: TBC domain; Blue: Rhodanese domain: Yellow: PH domain. WT: wild type; TR: TBC and Rhodanese domain; RC: Rhodanese and C terminal PH domain; C: C terminal PH domain; TBC1D23 3 K: TBC1D23 with a K632E/K633E/K634E triple mutant. **c** HEK293T cells expressed different truncates were subjected to immunoprecipitation using GFP beads. Bound TBK1 was analyzed by immunoblotting. **d** HEK293T cells were expressed GFP-TBK1 together with TBC1D23 WT-mCherry or 3K-mCherry and subjected to immunoprecipitation using GFP beads. Bound TBK1 was analyzed by immunoblotting. **e** MEFs stably expressing TBC1D23-targeting shRNA (sh*Tbc1d23*), or control shRNA (shCT) were rescued with an empty vector, TBC1D23 WT or 3 K mutant. Cells were treated with DMXAA (25 μg/mL) for 30 min, and then subjected to immunoblotting. *: Long time exposure. One representative experiment of at least three independent experiments is shown.

The above results suggest that TBC1D23 promotes the translocation of TBK1 from endosomes to the TGN to regulate STING-IFN-I signaling. To gain more insights into the process, we characterized the interaction between TBC1D23 and TBK1. First, we found that FAM21 robustly immunoprecipitated TBK1 and IRF3 (Supplementary Fig. S11a). Similarly, TBC1D23 also robustly co-immunoprecipitated with TBK1, and the interaction slightly increased upon diABZI treatment (Fig. 5a). TBK1 L693Q that was deficient in associating with membranes also decreased the interactions with TBC1D23 (Supplementary Fig. S11b). More importantly, the association between TBC1D23 and TBK1 was strongly disrupted by depletion of FAM21, indicating that FAM21 may mediate their interaction (Supplementary Fig. S11c). To determine which domain of TBC1D23 interacted with TBK1, we made a series of truncations^39–41^. The PH domain of TBC1D23 was the minimal domain that immunoprecipitated TBK1, and more TBK1 was retained by TBC1D23 RC, which harbors the Rhodanese domain in addition to the PH domain. In contrast, TBC1D23 TR, which harbors the TBC and Rhodanese domains, did not interact with TBK1 (Fig. 5b, c). Thus, the C-terminal PH domain of TBC1D23 is mainly responsible for contacting TBK1, and the Rhodanese domain further promotes the interaction.

We have previously identified a K632E/K633E/K634E triple mutant (termed as 3 K) within the C-terminal domain of TBC1D23, which was defective in FAM21 binding and in mediating endosome-to-TGN trafficking^39^. The same mutant also abolished the interaction with TBK1 (Fig. 5d). To determine how the TBC1D23-TBK1 interaction regulated STING signaling, we re-expressed TBC1D23 WT and 3 K in TBC1D23-depleted MEFs. Re-expression of TBC1D23 WT, but not 3 K, rescued both TBK1 and STING phosphorylation (Fig. 5e). Thus, TBC1D23, via its C-terminus, promotes the trafficking of TBK1 to the TGN and regulates the STING-IFN-I signaling.

Next, we examined whether TBC1D23 regulates STING trafficking and phosphorylation. Consistent with previous results, DMXAA treatment induced the translocation of STING to the Golgi. Deletion of *Tbc1d23* in MEFs using two different shRNAs did not alter the Golgi fraction of STING, indicating that TBC1D23 was dispensable for the translocation of STING from ER to the Golgi (Supplementary Fig. S12a, b). Deletion of *Tbc1d23*, however, drastically reduced the level of phosphorylated STING upon DMXAA treatment, which is consistent with reduced levels of TBK1 phosphorylation (Supplementary Fig. S12c, d). Thus, TBC1D23 is critical for the translocation of TBK1 to the Golgi, but not for STING.

## Discussion

**Fig. 6:**
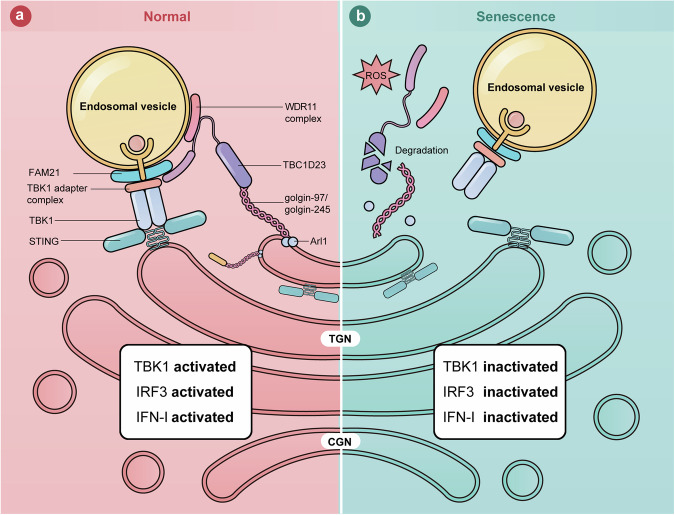
A model showing how TGN tethering factors regulate the STING-IFN-I signaling. **a** In normal cells, TGN tethering factors promote STING-IFN-I signaling by mediating endosome-to-TGN translocation of TBK1. **b** In senescent cells, stresses, such as ROS, lead to the degradation of TGN tethering factors. The endosome-to-TGN translocation of TBK1 is blocked, and STING-IFN-I signaling is inhibited.

Activation of the cGAS–STING pathway promotes two major downstream pathways: the production of IFN-I via IRF3, and pro-inflammatory responses through NF-κB^2^. How these two pathways downstream of cGAS–STING are differentially regulated is one of the major questions in the field. Here, we show that TGN tethering factors mainly regulate the STING-IFN-I signaling (Fig. 6a, b). Furthermore, we demonstrate that oxidative stress leads to the dislocation and degradation of the TGN tethering factors, resulting in the inhibition of the STING-IFN-I signaling. Altogether, our work reveals a mechanism that specifically regulates the STING-IFN-I signaling in senescent cells and helps to explain why aged individuals exhibit activated inflammation but are immunocompromised^66–68^.

How are the STING-IFN-I and STING-NF-κB signaling pathways differentially regulated? First, the two pathways are regulated by different upstream kinases. TBK1/IKKε are kinases known to be activated by STING to induce the production of IFN-I; in contrast, multiple kinases, in addition to TBK1/IKKε, including transforming growth factor beta-activated kinase 1 (TAK1), IkB kinase β (IKKβ), mitogen-activated protein kinase (MAPK) are known to mediate STING-regulated NF-κB activation^69,70^. Second, the two pathways are activated at different subcellular compartments. IRF3 is activated at the TGN by the signalosomes formed by STING and TBK1^71,72^; on the other hand, activation of NF-κB by STING can occur at multiple subcellular compartments. For instance, Stempel et al. found that STING induced NF-κB activation in the endoplasmic reticulum before being transported to the TGN^70,73^. Consistently, our studies demonstrate that the STING-IFN-I signaling, but not the STING-NF-κB signaling, highly depends on the TGN tethering factors.

Among the TGN tethering factors that we have characterized, golgin-97 seems to have functions opposite to the rest of them. For instance, deletion of golgin-245, TBC1D23, WDR11 complex and FAM21 all suppressed the STING-IFN-I signaling and did not dramatically affect the STING-NF-κB signaling. In contrast, deletion of golgin-97 activated both the IFN-I and NF-κB signaling. We suspect that golgin-97 may regulate the cGAS-STING pathway independent of its functions in vesicle tethering. Indeed, a recent report also observed that depletion of golgin-97 leads to activation of NF-κB^74^. The authors suggested that golgin-97 inhibits NF-κB activation by inhibiting IκBα degradation in a manner independent of the vesicular tethering function.

Together with previous studies, we have identified Arl1 as a key therapeutic target to modulate the IFN-I signaling. Oxidative stress promotes the degradation of Arl1, which in turn leads to the dislocation and degradation of multiple TGN tethering factors, suppressing the IFN-I signaling^46^. Thus, small molecules inhibiting the degradation of Arl1 may restore the levels of TGN tethering factors and enhance the STING-IFN-I signaling. We envision that these molecules could boost antiviral and antitumor functions in aged individuals, with insignificant effect on chronic inflammation mediated by NF-κB.

## Materials and methods

### Reagents and antibodies

Reagents used for this study were as follows: Vadimezan (DMXAA) (Topscience, T6273), diABZI (Selleck, S8796), Manganese (II) chloride tetrahydrate (Sangon Biotech, A500331), MnJβ (Mnstarter Bio, MS0001), Hydrogen peroxide solution (30%) (Sigma, 1.08597), Anti-GFP nanobody magarose beads (Alpalife Bio, KTSM1334), Etoposide (MCE, HY-13629), Doxycycline Hyclate (Yeasen, 60204ES03), Liposomal transfection reagent (Yeasen, 40802ES03), N-Acetyl-L-cysteine (MCE, HY-B0215), D-Biotin (Beyotime, ST2051), protease inhibitor cocktail (Selleck, B14001), phosphatase inhibitor cocktail (Selleck, B15001), DAPI (Servicebio, G1012), Streptavidin magnetic beads (Beyotime, P2151), Polybrene (Yeasen, 40804ES76), Poly I:C (Sigma, 31852-29-6), Bromobenzene (Aladdin, B103388-500g), Alloxan (Sigma, A7413), Rapamycin (MCE, HY-10219).

Antibodies used in this study were as follows: rabbit anti-TBC1D23 (Proteintech, 17002-1-AP, Western blot (WB) 1:1000), rabbit anti-FAM21 (donated by Dr. Daniel D. Billadeauh, WB 1:1000, IF 1:300), rabbit anti-golgin-97 (Proteintech, 12640-1-AP, IF 1:200, WB 1:1000), mouse anti-GFP (Proteintech, 66002-1-Ig, WB 1:2000), rabbit anti-GAPDH (Proteintech, 10494-1-AP, WB 1:2000), rabbit anti-beta actin (ABclonal, AC026, WB 1:2000), rabbit anti-mCherry (Proteintech, 26765-1-AP, WB 1:2000), rabbit anti-Phospho-TBK1/NAK (Ser172) (Cell Signaling Technology, 5483 T, WB 1:1000, IF 1:300), rabbit anti-Phospho-STING (Ser366) (Cell Signaling Technology, 50907 T, WB 1:1000, IF 1:300), rabbit anti-Phospho-IRF-3 (Ser396) (Cell Signaling Technology, 4947S, WB 1:1000), rabbit Phospho-NF-κB p65 (Ser536) (Cell Signaling Technology, 3033 T, WB 1:1000), rabbit anti-GOLGA4/golgin-245 (Cell Signaling Technology, 79145S, WB 1:1000), rabbit anti-WDR11 (abcam, ab93871, WB 1:1000), rabbit anti-IRF3 (abcam, ab68481, WB 1:1000), rabbit anti-STING (Proteintech, 19851-1-AP, WB 1:1000), mouse antiGM130 (BD, 610822, IF 1:300), rabbit anti-Alpha Tubulin (Proteintech, 11224-1-AP, WB 1:1000), rabbit anti-EEA1 (Cell Signaling Technology, 3288 T, WB 1:1000), rabbit anti-Arl1 (Proteintech, 16012-1-AP, WB 1:1000) rabbit anti-FAM91A1 (Proteintech, 27738-1-AP, WB 1:1000), rabbit anti-RAB7 (Cell Signaling Technology, 9367 T, WB 1:1000), rabbit anti-NAK/TBK1 (abcam, ab40676, WB 1:1000, IF 1:300), Goat anti-Rabbit IgG Secondary Antibody HRP conjugated (SAB, L3012-2), Goat anti-Mouse IgG Secondary Antibody HRP conjugated (SAB, L3032-2). HRP-conjugated Affinipure Goat anti-Rat IgG (H + L) (Proteintech, SA00001-15). Alexa-labeled secondary antibodies were from Invitrogen (1:2000).

### Plasmids

Homo sapiens TBC1D23 (1-699) full-length and truncations were amplified by PCR from HEK293T cell cDNA and cloned into the pLVX-neo. CDS of EGFP was PCR-amplified from pEGFP-C1 and inserted into pLVX-neo-TBC1D23 and pLVX-neo-TBC1D23 truncations. pcDH-sh*Tbc1d23* plasmids were a gift from Chen Lu laboratory (Sichuan University). Start and stop codons were mutated to ensure the completion of the open reading frame. TBC1D23 and TBK1 mutants were generated through site-directed mutagenesis (MCLAB). FYVE-FKBP and TBK1-FRP genes were synthesized by Sangon biotech and cloned into the pTSB-Tight-tetR-F2A-Puro vector. The GRIP sequence of golgin-97 was fused to the C-terminus of EGFP (synthesized by Sangon Biotech) and inserted into the pTSB-Tight-tetR-F2A-Puro vector (Transheep Bio, TSB005112-2). The miR30a-based knockdown plasmid was obtained by inserting different targeting sequences into the miR30a backbone (synthesized by Sangon Biotech); the integrated sequence was cloned into the pTSB-Tight-tetR-F2A-Puro vector (Transheep Bio, TSB005112-2). EGFP-FAM21 plasmid was a gift from the Daniel D. Billadeauh laboratory. EGFP-TBK1 plasmid was a gift from the Pinglong Xu laboratory. Turbo-TBK1 plasmid was obtained by inserting PCR-amplified TBK1 into pCMV-N-Flag-miniTurboID vector (Beyotime, D3034). RFP-FYVE, RFP-CD63, RFP-Lamp1 plasmids were gifts from Yang Ge laboratory (Chinese Academy of Sciences). Lentiviral packaging plasmids psPAX2 and pMD2.G purchased from Addgene (Addgene, 12260, 12259).

### Mice

Female and male mice were sex-matched and studied at 6–8 weeks of age. All mice were housed in specific pathogen-free conditions in the Laboratory Animal Center of West China Second University Hospital, Sichuan University in accordance with the Guide for the Care and Use of Laboratory Animals. Animal protocols were approved by the Experimental Animal Management and Ethics Committee of West China Second University Hospital, Sichuan University. *Tbc1d23* flox/flox mice with C57BL/6 background were kindly provided by Professor Lu Chen (Sichuan University), with one flox site at the intron between exons 2 and 3 and another flox site at the intron between exons 3 and 4. Rosa26-SA-CreERT2 mice and the wild-type C57BL/6 were generated by GemPharmatech Co. Ltd. The genotyping primers are listed in Supplementary Table S2.

### In vivo treatments

Eight-week-old male *Tbc1d23* (flox/flox) mice and *Tbc1d23* (flox/flox, Cre-ERT) (*Tbc1d23* KO) mice were injected intraperitoneally with saline or MnJβ (40 mg/kg, Mnstarter Bio, MS0001) or DMXAA (25 mg/kg, Topscience, T6273), respectively. 10 or 3 h after injection, sera were collected for cytokine detection by ELISA, and organs (lung and kidney) were collected for qRT-PCR analysis.

For bromobenzene-induced mice model, eight-week-old male C57BL6 mice were received a single bromobenzene (dissolved in corn oil) oral gavage at a dosage of 0.3 mL/kg body weight, as previously described in ref. ^48^. For alloxan-induced mice model, eight-week-old male C57BL6 mice were received a single alloxan (dissolved in saline) intraperitoneal injection at a dosage of 150 mg/kg body weight, as previously described in ref. ^49^. One week later, the mice were injected intraperitoneally with DMXAA (25 mg/kg). Three hours after injection, sera were collected for cytokine detection by ELISA, and the livers were collected for qRT-PCR analysis.

### Peritoneal macrophages

8–10week-old mice were intraperitoneally injected with 40 mg/kg of MnJβ adjuvant dissolved in saline. Mice were then euthanized after 10 h of injection. 10 mL of ice-cold PBS was injected into the peritoneal cavity followed by gently massaged for 1 min. The peritoneal fluid was collected using a 15 mL tube. After centrifugation at 1,500 rpm for 5 min, the cell pellets were suspended in lysis buffer (Solarbio) to discard the red blood cells. The resulting cells were collected for further analysis.

### Cell culture

HEK293T (ATCC) and COS7 (ATCC) cells were cultured in Dulbecco’s modified Eagle’s medium (DMEM, Gibco) supplemented with 10% fetal bovine serum (NEWZERUM) and 1% penicillin/streptomycin (Hyclone) at 37 °C with 5% CO2. THP-1 (ATCC) cells were cultured in Roswell Park Memorial Institute (RPMI) 1640 (1640, Gibco) supplemented with 20% fetal bovine serum (NEWZERUM) and 1% penicillin/streptomycin (Hyclone) at 37 °C with 5% CO_2_. HCMEC/D3 (ATCC) cells were cultured in Endothelial Cell Medium (Thermal) supplemented with 5% fetal bovine serum (NEWZERUM) and 1% penicillin/streptomycin (Hyclone) at 37 °C with 5% CO_2_.

### Lentiviral production and infection

Transient transfection of plasmids in HEK293T cells was performed using Liposomal transfection reagent (Yeasen) according to the manufacturer’s protocol. For lentiviral packaging and infection, psPAX2 and pMD2.G plasmids were co-transfected with lentivirus vectors into 293T cells. The supernatant of the medium was collected after 48 h and filtered by 0.45 µm pore size filter membrane. Cells were infected with virus-containing supernatants and polybrene (10 μg/mL) for 12 h. Cells that continued to be cultured for 72 h were screened with puromycin.

### Generation of *Tbk1/Ikkε* knockout cell line

The single guide RNAs targeting mouse *Tbk1* or *Ikkε* were inserted into the lentiCRISPRv2 vector (Supplementary Table S2). Constructs encoding Cas9 and gRNA were co-transfected with viral packaging plasmids (pMD2.G:psPAX2:v2/sgTBC1D23 = 1:2:4) into HEK293T cells using PEI. After 48 h, the viral supernatants were collected and filtered. The supernatant was used to infect B16 cells with 4 μg/mL polybrene (YEASEN). Cells were cultured in selection medium containing 5 μg/mL puromycin (BBI Life Sciences, A610593) for two generations, then trypsinized and diluted into 96-well plates. Single colonies were expanded and analyzed by immunoblotting.

### Immunofluorescence and live cell imaging

For immunofluorescence, cells were fixed using 4% paraformaldehyde and permeabilized with 0.1% (v/v) Triton X-100 in PBS, then blocked with goat serum in 5% BSA for 1 h. Cells were then incubated with indicated primary antibodies overnight at 4 °C. After washing three times with PBS, cells were incubated with the appropriate Alexa-labeled secondary antibodies at room temperature for 1 h. Cells were mounted, and confocal images were acquired using an Olympus IXplore SpinSR microscope (100× oil objective, NA = 1.45). X, Y scans were acquired at 8 µs/pixel with 1024 × 1024 resolution (sequential mode: line). Image acquisition and analysis within a certain set of experiments were performed with the same parameters. For each condition, the number of cells analyzed was indicated in the figure legends.

For live-cell imaging, HCMED/D3 cells were seeded onto 24-well glass-bottom dishes and then transfected with plasmids encoding EGFP-GRIP. After 24 h, cells were stimulated with H_2_O_2_, and images were acquired using a Spinning Disk Confocal microscope (Olympus SpinSR10) at the indicated time. For live cell imaging of TBK1 distribution on vesicles, COS7 cells cultured on 24-well glass-bottom dishes were co-transfected with EGFP-TBK1 and vesicle markers (mCherry-FYVE, mCherry-CD63 or mCherry-Lamp1) for 24 h, and images were captured using Olympus SpinSR10. COS7 cells cultured on 24-well glass-bottom dishes were co-transfected with plasmids encoding EGFP-TBK1, TBC1D23-mCherry and BFP-FYVE for 24 h. *Tbk1*/*Ikkε* KO B16 cells were cultured on 24-well glass-bottom dishes. The cells were co-transfected with plasmids encoding EGFP-TBK1 WT or L693Q, together with mCherry-FYVE for 24 h. Images were acquired using Olympus SpinSR10.

### Primary cells

For BMDMs preparation, femurs and tibias were dissected from *Tbc1d23* (flox/flox) mice and *Tbc1d23* (flox/flox, Cre-ERT) mice. Bone marrow was flushed from the femurs and tibias, and the red blood cells were lysed with lysis buffer (Solarbio). Debris was removed by passing cells through a 70 μM strainer and cells were cultured in 10 cm dishes in DMEM supplemented with 20% fetal bovine serum (FBS) plus 30% L929 conditional medium for BMDM differentiation. The medium was changed after 4 days. On day 6, cells were collected for subsequent experiments.

For primary mouse lung fibroblasts (MLFs) preparation, lungs isolated from C57BL/6 mice were minced and digested in magnesium and calcium-free HBSS buffer supplemented with 5 μL/mice DNase I (Beyotime) and 10 mg/mL type I collagenase (YEASEN) for 3 h at 37 °C. The lung pieces were blown to liquid and then centrifuged at 1500 rpm for 5 min, and the cell pellets were cultured in DMEM supplemented with 15% FBS and 1% streptomycin–penicillin. After two days, adherent fibroblasts were washed with PBS and cultured for various experiments.

### Immunoprecipitation

THP-1 cells were infected with lentivirus encoding the miR30a-based targeting sequence and screened with puromycin. Afterward, cells were cultured for 72 h in medium with or without 1 μg/mL of doxycycline. A fraction of cells was harvested and lysed with 2× SDS loading buffer to determine the knockdown efficiency. Samples were separated by SDS-PAGE and detected by immunoblotting with the indicated antibodies. The remaining cells were counted and used for immunostimulation.

### Doxycycline-induced gene knockdown

THP-1 cells were infected with lentivirus encoding the miR30a based targeting sequence and screened with puromycin. Afterward, cells were cultured for 72 h in medium with or without 1 μg/mL of doxycycline, respectively. A fraction of cells was harvested lysed with 2× SDS loading buffer to determine the knockdown efficiency. Samples were separated by SDS-PAGE and detected by immunoblotting with the indicated antibodies. The remaining cells were counted and used for immunostimulation.

### Cell immunostimulation

THP-1 cells were induced with PMA for 24 h and then cultured with 2% serum for 24 h before immune stimulation. THP-1 cells were cultured in medium with or without 200 μM MnCl_2_ for 20 h. Afterward, cells were collected by centrifugation for subsequent analysis. THP-1 cells were treated with 2 μg/mL of Poly I:C (liposome transfection) for 4 h. Cells were collected by centrifugation for subsequent analysis. THP-1 cells were treated with 25 μM diABZI for 1 h and then centrifuged to harvest the cells for subsequent analysis.

### Immunoblotting

Cells were harvested by centrifugation, and cell pellets were resuspended in 1× SDS sample buffer (62.5 mM Tris-HCl, pH 6.8, 2% SDS, 20 mM DTT and 10% glycerol), sonicated, and boiled at 95 °C for 10 min. Proteins were separated by SDS-PAGE and blotted on a 0.22 μm polyvinylidene difluoride (PVDF) membrane (Millipore). Immunoblots were blocked with 5% (w/v) milk in TBST (0.1% (v/v) Tween-20 in TBS) at room temperature for 1 h then incubated overnight at 4 °C with specific primary antibody followed by detection with HRP-conjugated secondary antibody. Bands were visualized by chemiluminescence (Abkam).

### Cellular senescence and oxidative stress induction

For the induction of cellular senescence, primary mouse lung fibroblasts were treated with 50 μM etoposide for 24 h. Alternatively, the cells were treated with 200 μM H_2_O_2_ for 2 h (serum-free) and then continued to be cultured for 24 h in complete medium.

### Quantitative real-time PCR and ELISA

Total RNA was extracted from the indicated cells using FastPure Complex Cell/Tissue Total RNA Isolation Kit (Vazyme) and reverse transcribed into cDNA with a 1st Strand cDNA Synthesis Super Mix Kit (YEASEN). Relative gene expression was then analyzed by qPCR using Universal Blue qPCR SYBR Master Mix (YEASEN) on a qTOWER³G Real-Time Thermal Cyclers (analytik jena). The primers used in this study are listed in Supplementary Table S1. The ELISA was performed using mouse IFN-β ELISA kit (BioLegend) and IL-6 ELISA kits (Dakewe) according to the manufacturers’ instructions.

### Isolation of endosome and heavy organelles fractions

Minute^TM^ Endosome Isolation and Cell Fractionation Kit (ED-028, Invent Biotechnologies) was used to enrich endosome fractions and heavy organelles fractions, as previously described. Briefly, 10 cm dish MEFs stably transfected pcDH-shCT or pcDH-sh*Tbc1d23* grown to 90%–95% confluence was collected, washed once with cold PBS, and suspended in 600 μL buffer A. Vortex the tube vigorously for 30 s. The cell suspension was then loaded into the filter cartridge, centrifuged at 16,000× *g* for 30 s. The supernatants were transferred to a fresh 1.5 mL tube and centrifuged for 2 min at 700× *g* at 4 °C. Then the supernatants were transferred to a fresh 1.5 mL tube and centrifuged for 30 min at 16,000× *g* at 4 °C. The pellets containing heavy organelles fractions were resuspended in 200 μL 1× SDS sample buffer, boiled for 10 min at 98 °C. The supernatant was transferred to a fresh 1.5 mL tube, and an equal volume of buffer B was added to the tube. The tube was incubated on ice for 1 h and then centrifuged at 10, 000× *g* for 1 h at 4 °C. The pellets containing endosome fractions were resuspended in 200 μL 1× SDS sample buffer, boiled for 10 min at 98 °C. The samples were separated by SDS-PAGE and detected by immunoblot with indicated antibodies.

Cell fractionation was performed as described previously^46^. Briefly, B16 cells were washed with PBS, collected in CF buffer (ED-028, Invent Biotechnologies) with a cell scraper. The cell suspension was then loaded into the filter cartridge (ED-028, Invent Biotechnologies), centrifuged at 16,000× *g* for 30 s. The supernatants were transferred to a fresh 1.5 mL tube and centrifuged for 2 min at 700× *g* at 4 °C. The supernatants were collected as the post nuclear supernatant (PNS) fraction. PNS was then subjected to ultracentrifugation for 1 h at 40,300 rpm (100,000× *g*) at 4 °C to separate membranes from cytosol.

### Statistics and reproducibility

Statistical analyses were performed using GraphPad Prism 8.0 software. No statistical methods were used to predetermine sample size. Each dataset was subjected to the D’Agostino & Pearson test, the Shapiro-Wilk test, or the Kolmogorov-Smirnov test for normal distribution (where applicable). For all experiments, statistical significance of differences between two groups of normally distributed data was tested using the unpaired two-tailed Student’s *t*-test. The unpaired two-tailed Mann-Whitney test was used to determine significance between two groups of non-normally distributed data. Statistical significance of differences between multiple groups of normally distributed data. Differences were considered significant when *p* < 0.05. All data are expressed as mean ± SD.

## Supplementary information

Supplementary figures

Supplementary Video S1

Supplementary Video S2
