# Supplementary material for: Trans-Golgi network tethering factors regulate TBK1 trafficking and promote the STING-IFN-I pathway
Source: Cell Discov. 2025 Mar 18;11:23. doi: 10.1038/s41421-024-00763-z (PMC11914254; doi:10.1038/s41421-024-00763-z)
Supplement: Supplementary file 1 — Supplementary figures [file 41421_2024_763_MOESM1_ESM.pdf]

Supplementary information

Supplementary Fig S1 | Certain TGN vesicle tethering factors are reduced in aged mice or upon oxidative stress.

Supplementary Figure S2 | STING-IFN-I signaling is significantly inhibited in oxidative stress mice.

Supplementary Figure S3 | TGN vesicle tethering factors are dispensable for activation of RIG-I-IFN-I signaling.

Supplementary Figure S4 | Deletion of TBC1D23 predominately impairs the STING-IFN-I signaling.

Supplementary Figure S5 | Distribution of TBK1 in different vehicles.

Supplementary Figure S6 | FAM21 co-localized with TBK1 at endosomes.

Supplementary Figure S7 | STING activation induces TBK1 endosome-to-TGN translocation.

Supplementary Figure S8 | TBC1D23 promotes TBK1 phosphorylation at the Golgi apparatus.

Supplementary Figure S9 | Endosomal localization of TBK1 is critical for the STING signaling.

Supplementary Figure S10 | Endosomal targeting of TBK1 enhances the STING signaling activation.

Supplementary Figure S11 | FAM21 interacts with TBK1 and IRF3 and mediates the binding of TBC1D23 to TBK1.

Supplementary Figure S12 | TBC1D23 is indispensable for STING phosphorylation, but not for STING translocation to Golgi.

Supplementary Table S1 | Sequence of qPCR primers used in this study.

Supplementary Table S2 | Sequence of gene knock down target used in this study.

Supplementary Video S1 | Live cell imaging of COS7 cells overexpressing RFP-FYVE (red) and EGFP-TBK1 (green).

Supplementary Video S2 | Live cell imaging of COS7 cells overexpressing BFP-FYVE (blue), EGFP-TBK1 (green) and TBC1D23-mCherry (red).

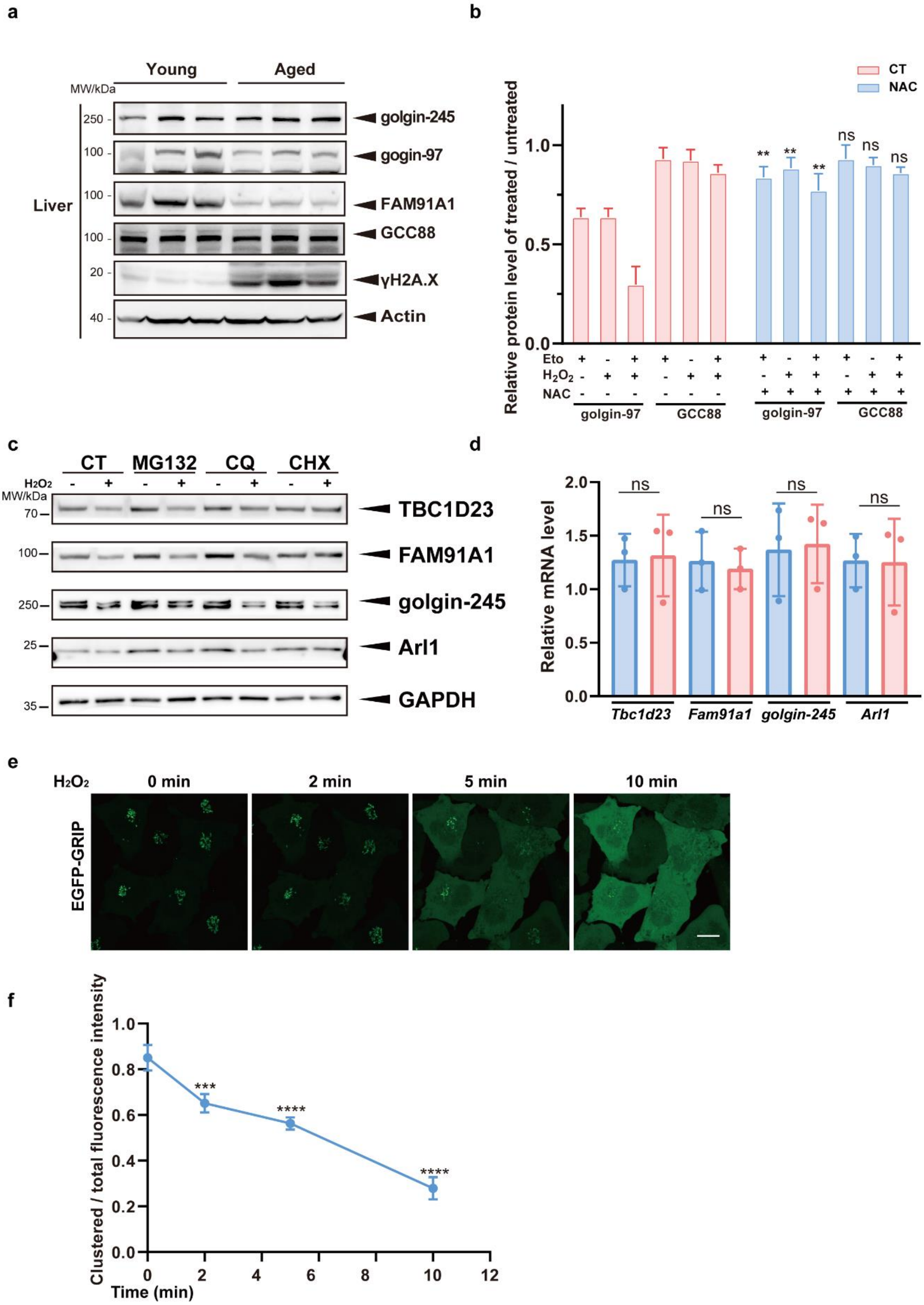
a
Young
Aged
MW/kDa
Liver
250
100
100
100
20
40
golgin-245
gogin-97
FAM91A1
GCC88
γH2A.X
Actin
b
CT
NAC
Relative protein level of treated / untreated
1.0
0.5
0.0
** ** ** ns ns ns
Eto
H2O2
NAC
golgin-97
GCC88
golgin-97
GCC88
c
CT
MG132
CQ
CHX
H2O2
MW/kDa
70
100
250
25
35
TBC1D23
FAM91A1
golgin-245
Arl1
GAPDH
d
Relative mRNA level
2.0
1.5
1.0
0.5
0.0
ns
ns
ns
ns
Tbc1d23
Fam91a1
golgin-245
Arl1
e
H2O2
0 min
2 min
5 min
10 min
EGFP-GRIP
f
Clustered / total fluorescence intensity
1.0
0.8
0.6
0.4
0.2
0.0
***
****
****
0
2
4
6
8
10
12
Time (min)

Supplementary Figure S1 | Certain TGN vesicle tethering factors are reduced in aged mice or upon oxidative stress.

(a) Livers from young and aged mice were extracted and analyzed for expression levels of TGN vesicle tethering factors by immunoblotting.

(b) Primary mouse lung fibroblasts were treated with etoposide (50 μM) for 12 h or pretreated with H_2O_2 (200 μM) for 2 h and continued to be cultured in the presence or absence of etoposide (50 μM) for 12 h. Untreated cells were used as a control, and the trans-Golgi vesicle tethering factors levels were analyzed by immunoblotting. Statistical analysis of golgin-97 and GCC88 expression levels. The levels were determined by normalizing the image gray values in Fig. 1c.

(c) Primary mouse lung fibroblasts were first treated with MG132, CQ, or CHX for 1 h, followed by treatment with H_2O_2 (500 μM) for 2 h. Untreated cells were used as a control, and the protein levels of TGN tethering factors were analyzed by immunoblotting.

(d) Primary mouse lung fibroblasts were treated with H_2O_2 (500 μM) for 2 h. Untreated cells were used as a control, and mRNA levels of TGN tethering factors were analyzed by qPCR.

(e) Time-lapse live cell imaging of A549 cells stably expressing EGFP-GRIP with H_2O_2 (500 μM). Scale bar = 5 μm.

(f) Quantifying the ratio of clustered protein fluorescence intensity to total fluorescence intensity within a cell (10 cells, average). Three biological replicates were performed.

One representative experiment of at least three independent experiments is shown. Data analyzed by two-tailed t-test and shown as mean ± SD ($n \geqslant 3$). ns, not significant, $p > 0.05$; * $p < 0.05$; ** $p < 0.01$; *** $p < 0.001$; **** $p < 0.0001$.

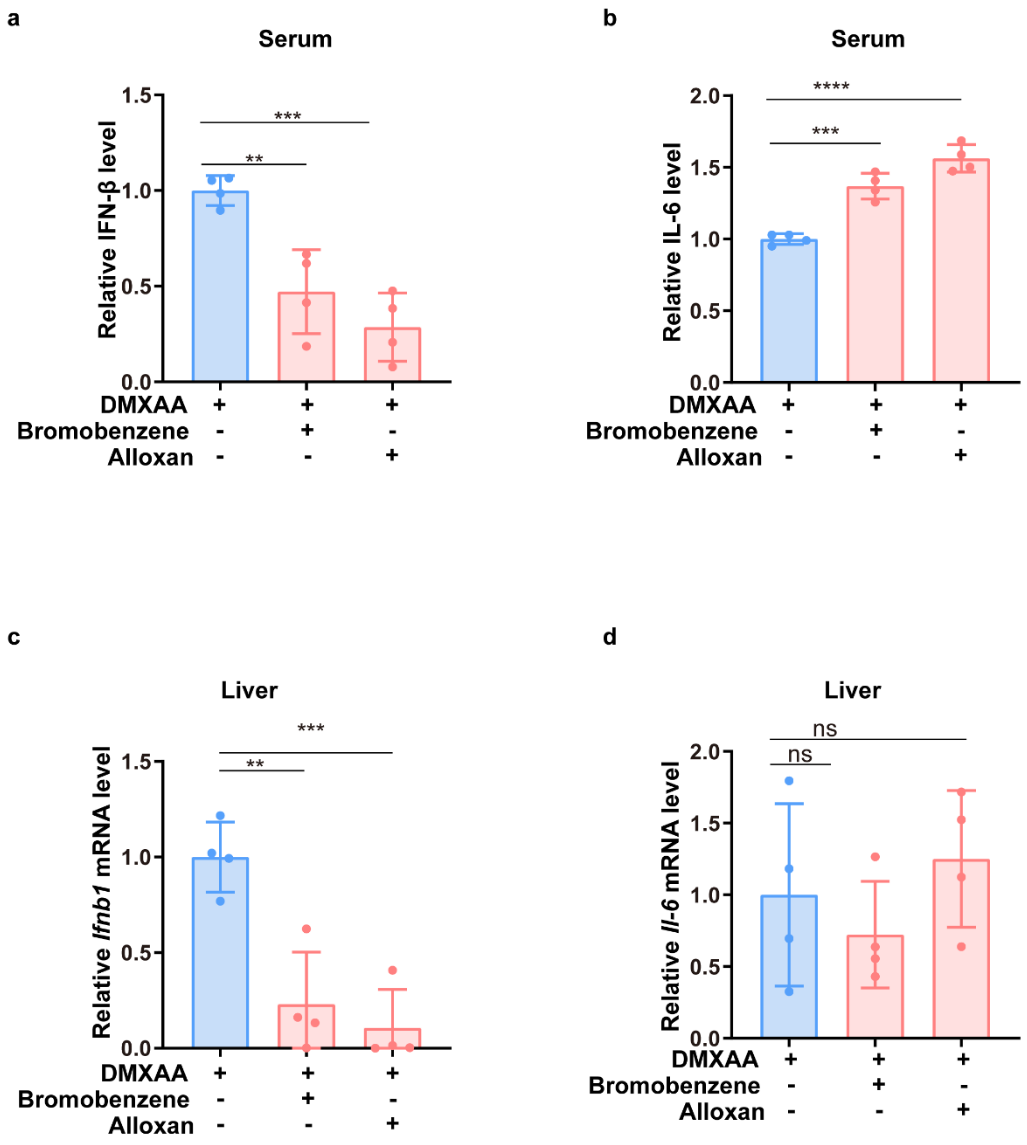

a
Serum
Relative IFN-β level
1.5
1.0
0.5
0.0
***
**
DMXAA + + +
Bromobenzene - + -
Alloxan - - +
b
Serum
Relative IL-6 level
2.0
1.5
1.0
0.5
0.0
****
***
DMXAA + + +
Bromobenzene - + -
Alloxan - - +
c
Liver
Relative *Ifnb1* mRNA level
1.5
1.0
0.5
0.0
***
**
DMXAA + + +
Bromobenzene - + -
Alloxan - - +
d
Liver
Relative *Il-6* mRNA level
2.0
1.5
1.0
0.5
0.0
ns
ns
DMXAA + + +
Bromobenzene - + -
Alloxan - - +

Supplementary Figure S2 | STING-IFN-I signaling is significantly inhibited in oxidative stress mice.

(a-b) Mice were injected intragastrically with 0.3 mL/kg of bromobenzene or intraperitoneally with 150 mg/kg of alloxan. Injection of an equal amount of solvent was as a control. One week later, the mice were injected intraperitoneally with 25 mg/kg of DMXAA (5 mg/mL, dissolved in 7.5% NaHCO3). Three hours later, the levels of serum IFN-β (a) and IL-6 (b) were determined with an ELISA kit.

(c-d) Mice were treated as (a-b). Three hours after DMXAA treatment, livers from each mouse were extracted, and the expression level of *Ifnb1* (c) and *Il-6* (d) was determined by qPCR. One representative experiment of at least three independent experiments is shown. Data analyzed by two-tailed t-test and shown as mean ± SD (n ≥ 3). ns, not significant, $p > 0.05$; * $p < 0.05$; ** $p < 0.01$; *** $p < 0.001$; **** $p < 0.0001$.

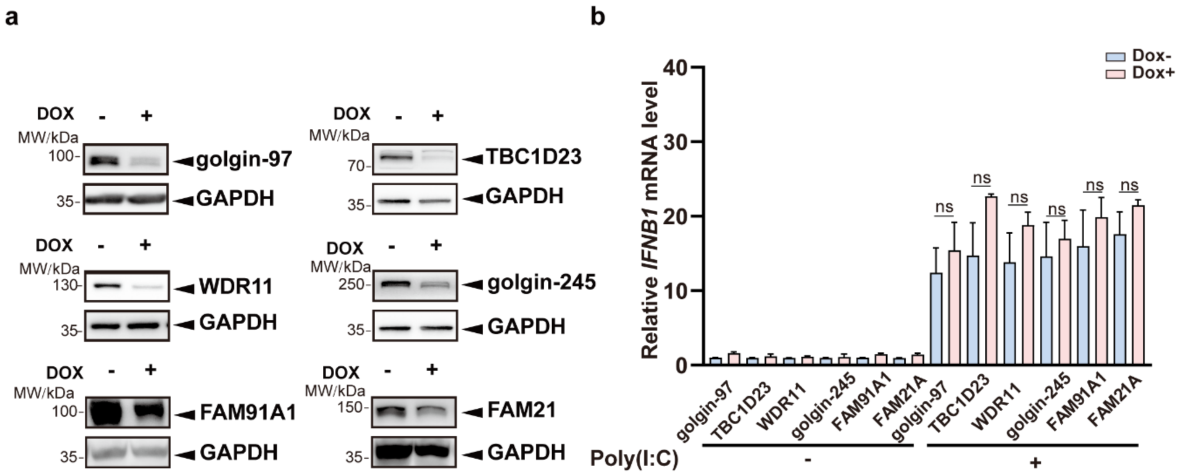
a
DOX - +
MW/kDa
100-
golgin-97
35-
GAPDH
DOX - +
MW/kDa
70-
TBC1D23
35-
GAPDH
DOX - +
MW/kDa
130-
WDR11
35-
GAPDH
DOX - +
MW/kDa
250-
golgin-245
35-
GAPDH
DOX - +
MW/kDa
100-
FAM91A1
35-
GAPDH
DOX - +
MW/kDa
150-
FAM21
35-
GAPDH
b
Dox-
Dox+
Relative IFNB1 mRNA level
40
30
20
10
0
ns
golgin-97
TBC1D23
WDR11
golgin-245
FAM91A1
FAM21A
Poly(I:C)
-
+

Supplementary Figure S3 | TGN vesicle tether are dispensable for activation of RIG-I-IFN-I signaling.

(a) THP-1 cells stably expressing an inducible gene knockdown system were treated with dox (1 μM) for 72 h. Gene knockdown effects were analyzed by immunoblotting.

(b) Cells in (a) were treated with Poly(I:C) (2 μg / mL) for 6 h and analyzed for *IFNB1* expression by qPCR.

One representative experiment of at least three independent experiments is shown. Data analyzed by two-tailed t-test and shown as mean ± SD ($n \geq 3$). ns, not significant, $p > 0.05$; * $p < 0.05$; ** $p < 0.01$; *** $p < 0.001$; **** $p < 0.0001$.

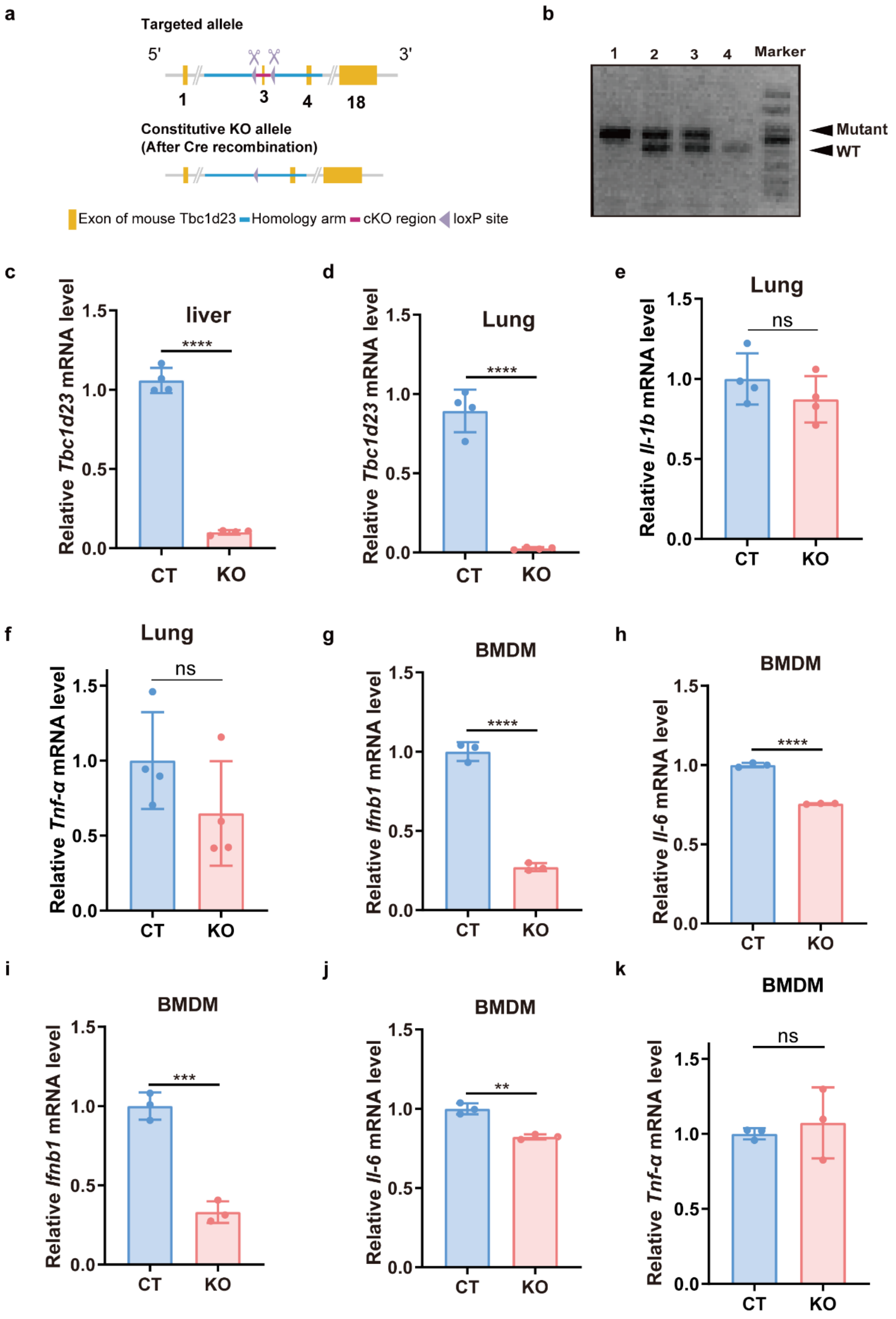

a
Targeted allele
5'
3'
1
3
4
18
Constitutive KO allele
(After Cre recombination)
Exon of mouse Tbc1d23
Homology arm
cKO region
loxP site
b
1
2
3
4
Marker
Mutant
WT
c
liver
Relative Tbc1d23 mRNA level
****
CT
KO
d
Lung
Relative Tbc1d23 mRNA level
****
CT
KO
e
Lung
Relative Il-1b mRNA level
ns
CT
KO
f
Lung
Relative Tnf-α mRNA level
ns
CT
KO
g
BMDM
Relative Ifnb1 mRNA level
****
CT
KO
h
BMDM
Relative Il-6 mRNA level
****
CT
KO
i
BMDM
Relative Ifnb1 mRNA level
***
CT
KO
j
BMDM
Relative Il-6 mRNA level
**
CT
KO
k
BMDM
Relative Tnf-α mRNA level
ns
CT
KO

Supplementary Figure S4 | Deletion of TBC1D23 predominately impairs the STING-IFN-I signaling.

(a) Schematic structure and experiment design of *Tbc1d23* knockout mice.

(b) Representative results of mouse genotype identification by PCR.

(c-d) Organs from each mouse were extracted, and the expression level of *Tbc1d23* was determined by qPCR.

(e-f) CT or *Tbc1d23* KO mice were injected intraperitoneally with 25 mg/kg of DMXAA (5 mg/mL) dissolved in 7.5% NaHCO3. Three hours later, organs from each mouse were extracted, and the expression level of *Il-1b* (e) and *Tnf-α* (f) was determined by qPCR.

(g-h) BMDM cells obtained from CT or *Tbc1d23* KO mice were treated with $MnCl_2$ (200 μM) for 20h, the expression levels of *Ifnb1* (e), *Il-6* (f) were analyzed by qPCR.

(i-k) BMDM cells were treated with DMXAA (25 μg/mL) and analyzed for expression levels of *Ifnb1* (g), *Il-6*(h), *Tnf-α* (i) by qPCR.

One representative experiment of at least three independent experiments is shown. Data analyzed by two-tailed t-test and shown as mean ± SD ($n \geq 3$). ns, not significant, $p > 0.05$; * $p < 0.05$; ** $p < 0.01$; *** $p < 0.001$; **** $p < 0.0001$.

a

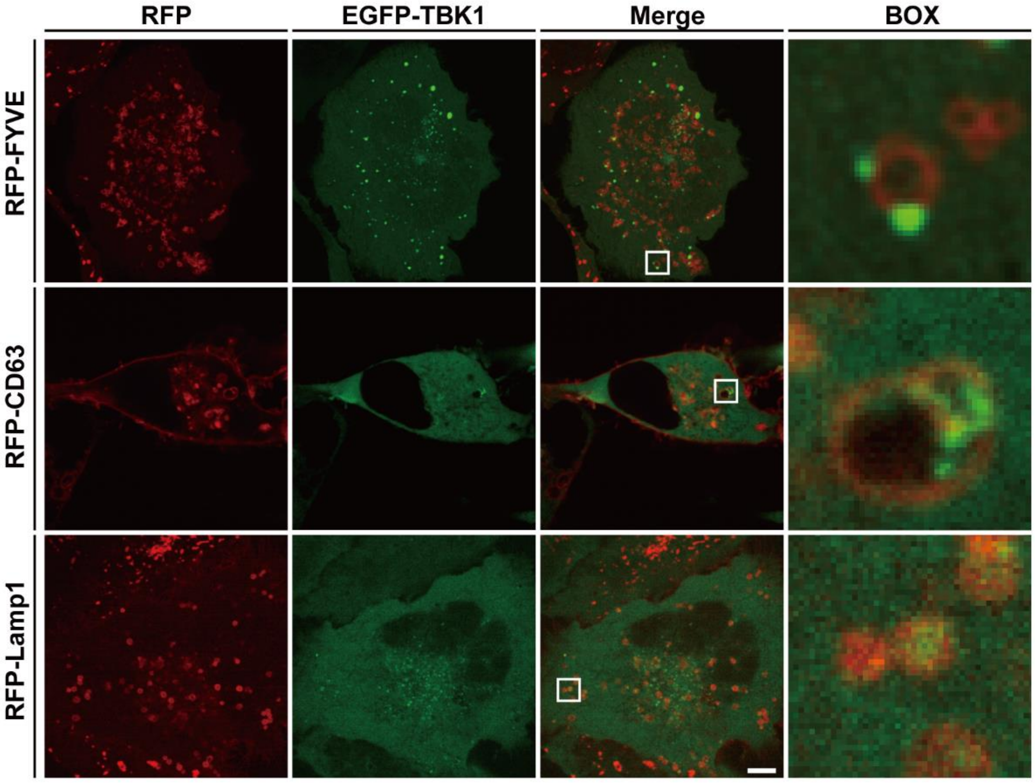

Supplementary Figure S5 | Distribution of TBK1 in different vehicles.

(a) COS7 cells expressing EGFP- TBK1 and RFP-tagged marker proteins were analyzed by confocal fluorescence microscopy. Scale bar = 5 μm.

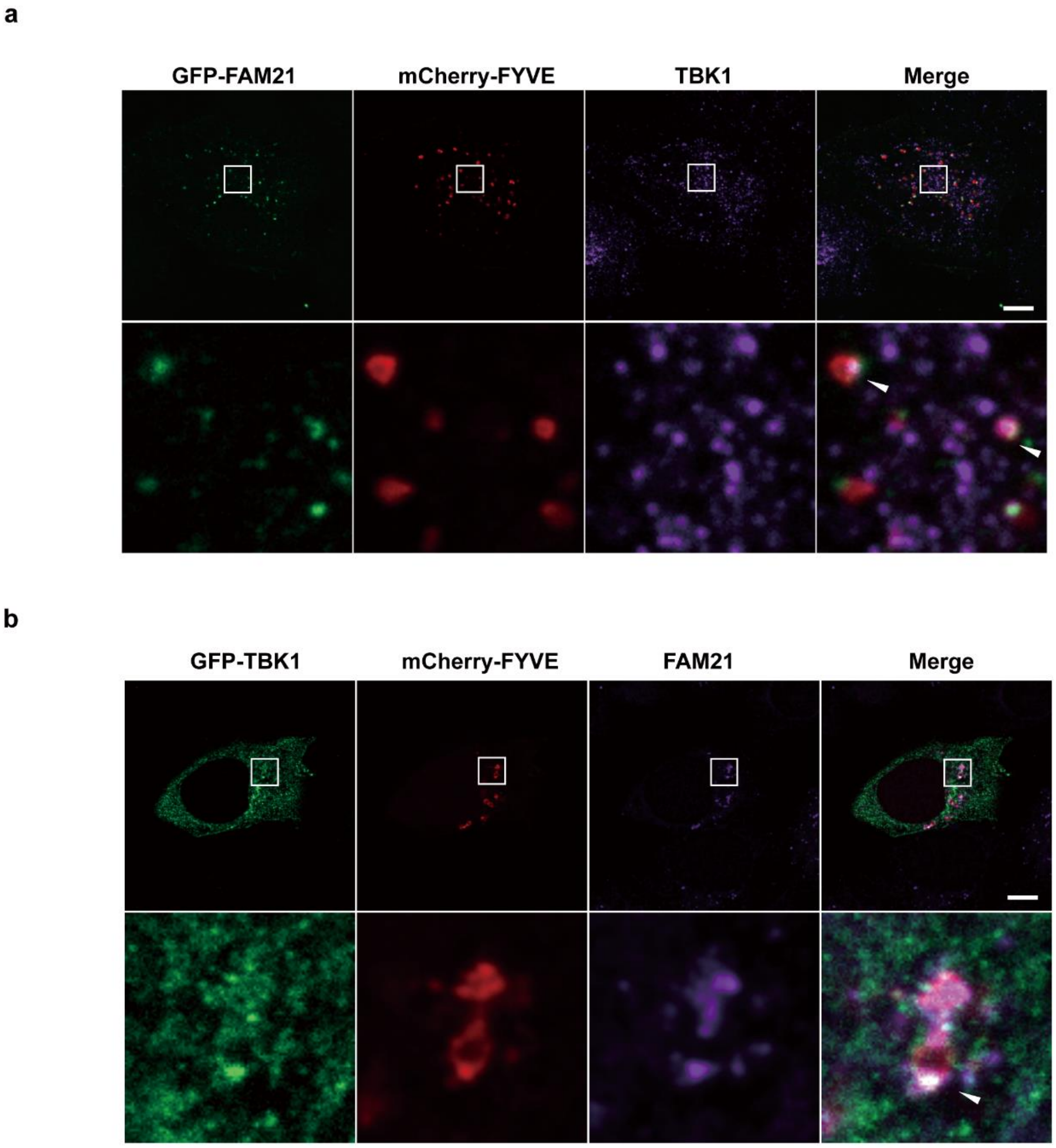
a
GFP-FAM21
mCherry-FYVE
TBK1
Merge
b
GFP-TBK1
mCherry-FYVE
FAM21
Merge

Supplementary Figure S6 | FAM21 co- localized with TBK1 at endosomes.

(a) GFP-FAM21 and mCherry-FYVE were expressed in A549 cells and endogenous TBK1 was labeled with an antibody; co-localization of GFP-FAM21 with TBK1 on endosomes labeled by mCherry-FYVE was analyzed by fluorescence confocal microscopy. Scale bar = 5 μm.

(b) GFP-TBK1 and mCherry-FYVE were expressed in A549 cells and endogenous FAM21 was labeled with an antibody; co-localization of GFP-TBK1 with FAM21 on endosomes labeled by mCherry-FYVE was analyzed by fluorescence confocal microscopy. Scale bar = 5 μm.

One representative experiment of at least three independent experiments is shown.

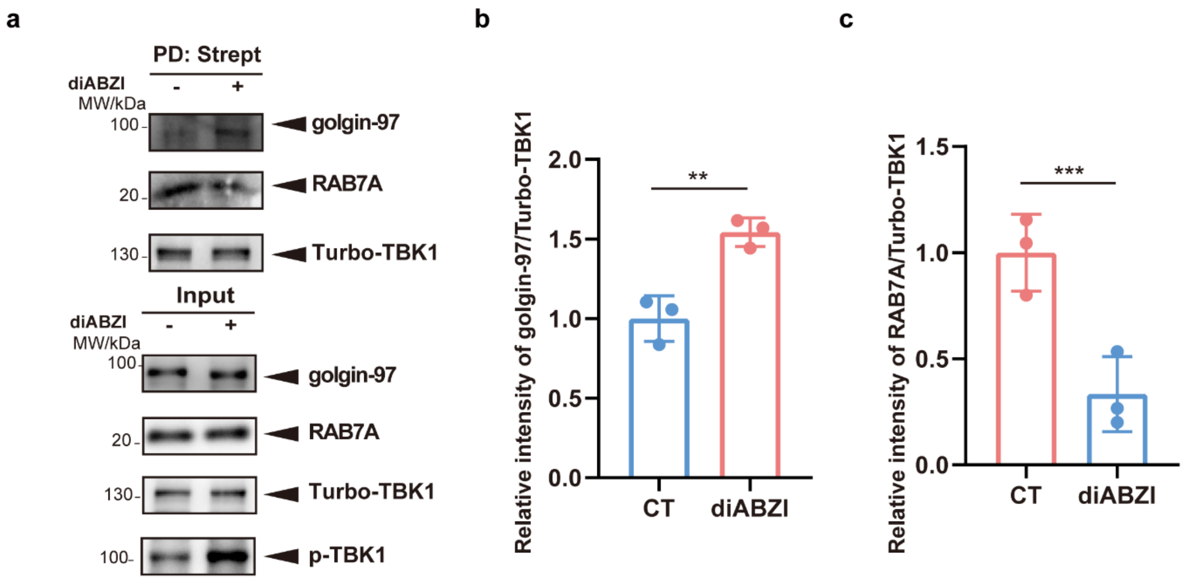

a
PD: Strept
diABZI - +
MW/kDa
100
golgin-97
20
RAB7A
130
Turbo-TBK1
Input
diABZI - +
MW/kDa
100
golgin-97
20
RAB7A
130
Turbo-TBK1
100
p-TBK1
b
Relative intensity of golgin-97/Turbo-TBK1
2.0
1.5
1.0
0.5
0.0
**
CT
diABZI
c
Relative intensity of RAB7A/Turbo-TBK1
1.5
1.0
0.5
0.0
***
CT
diABZI

Supplementary Figure S 7 | STING activation induces TBK1 endosome-to-TGN translocation.

(a) A549 cells stably expressing Turbo-TBK1 were stimulated with 2.5 μM diABZI for 30 min. After stimulation, biotin (500 μM) was added to the medium for 30 min, and the cell lysate was subjected to biotin pull-down using streptavidin beads. The bound samples were detected by immunoblotting. RAB7A and GOLGA1 were used to indicate endosomes/lysosome and TGN, respectively.

(b) Statistical analysis of biotin marked GOLGA1. The levels were determined by normalizing the image gray values in (a).

(c) Statistical analysis of biotin marked RAB7A. The levels were determined by normalizing the image gray values in (a).

One representative experiment of at least three independent experiments is shown. Data analyzed by two-tailed t-test and shown as mean ± SD ($n \geqslant 3$). ns, not significant, $p > 0.05$; * $p < 0.05$; ** $p < 0.01$; *** $p < 0.001$; **** $p < 0.0001$.

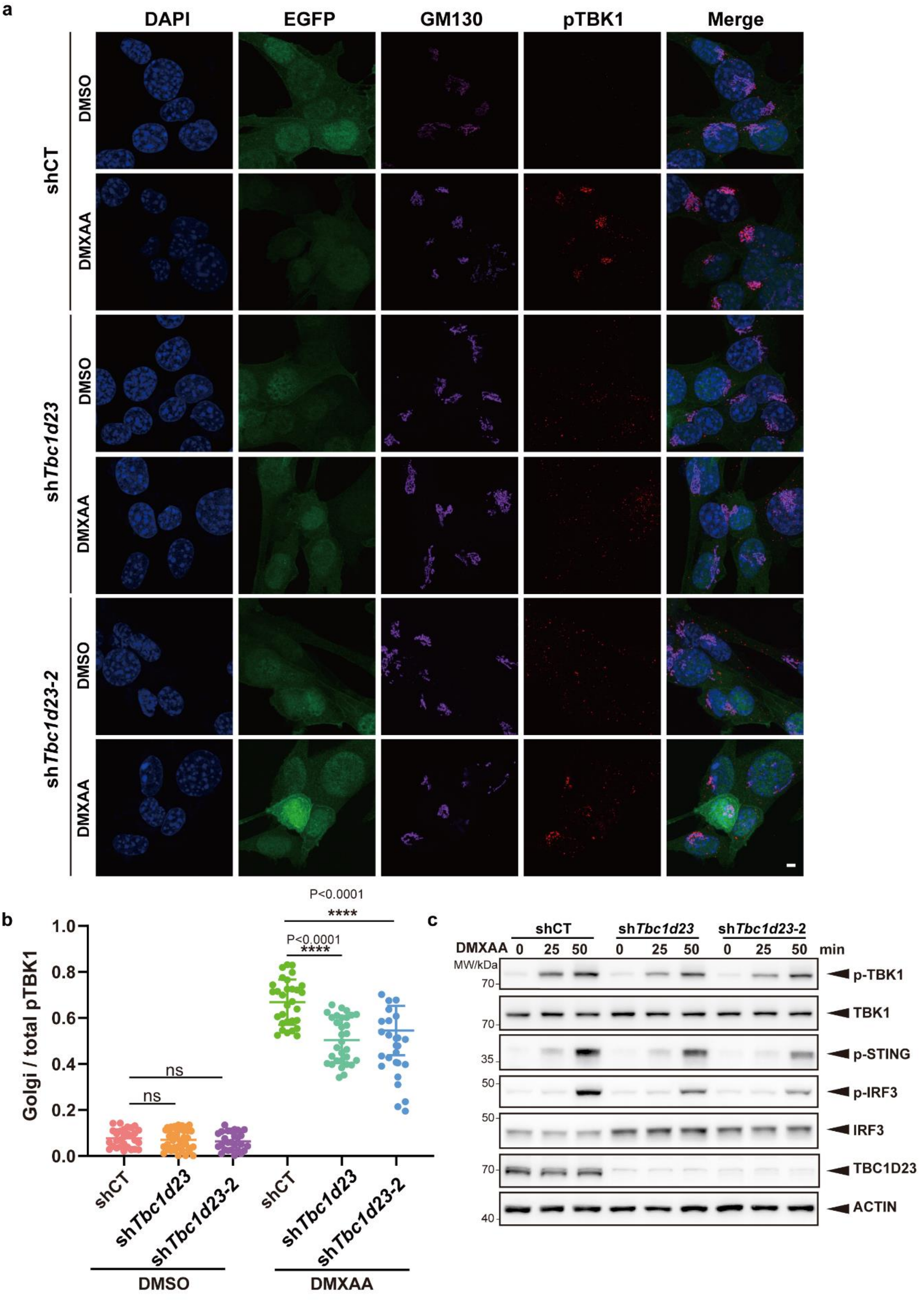

a
DAPI
EGFP
GM130
pTBK1
Merge
shCT
DMSO
DMXAA
shTbc1d23
DMSO
DMXAA
shTbc1d23-2
DMSO
DMXAA
b
Golgi / total pTBK1
1.0
0.8
0.6
0.4
0.2
0.0
ns
ns
P<0.0001
****
P<0.0001
****
shCT
shTbc1d23
shTbc1d23-2
shCT
shTbc1d23
shTbc1d23-2
DMSO
DMXAA
c
shCT
shTbc1d23
shTbc1d23-2
DMXAA 0 25 50 0 25 50 0 25 50 min
MW/kDa
70
p-TBK1
70
TBK1
35
p-STING
50
p-IRF3
50
IRF3
70
TBC1D23
40
ACTIN

Supplementary Figure S8 | TBC1D23 promotes TBK1 phosphorylation at the Golgi apparatus.

(a) MEFs knock down TBC1D23 by stably expressed shRNA (sh*Tbc1d23*, sh*Tbc1d23*-2), cells expressing untargeted shRNA served as a control (shCT). Cells were treated with DMXAA (25 μg/mL) for 30 min. Golgi-association of pTBK1 was analyzed by confocal fluorescence microscopy, using Golgi-resident protein GM130 as a reference. Scale bar = 5 μm.

(b) Quantitative analysis (a) of the ratio of Golgi pTBK1 to total pTBK1.

(c) Levels of pTBK, pSTING, and pIRF3 were analyzed by immunoblotting.

One representative experiment of at least three independent experiments is shown. Data analyzed by two-tailed t-test and shown as mean ± SD ($n \geq 3$). ns, not significant, $p > 0.05$; * $p < 0.05$; ** $p < 0.01$; *** $p < 0.001$; **** $p < 0.0001$.

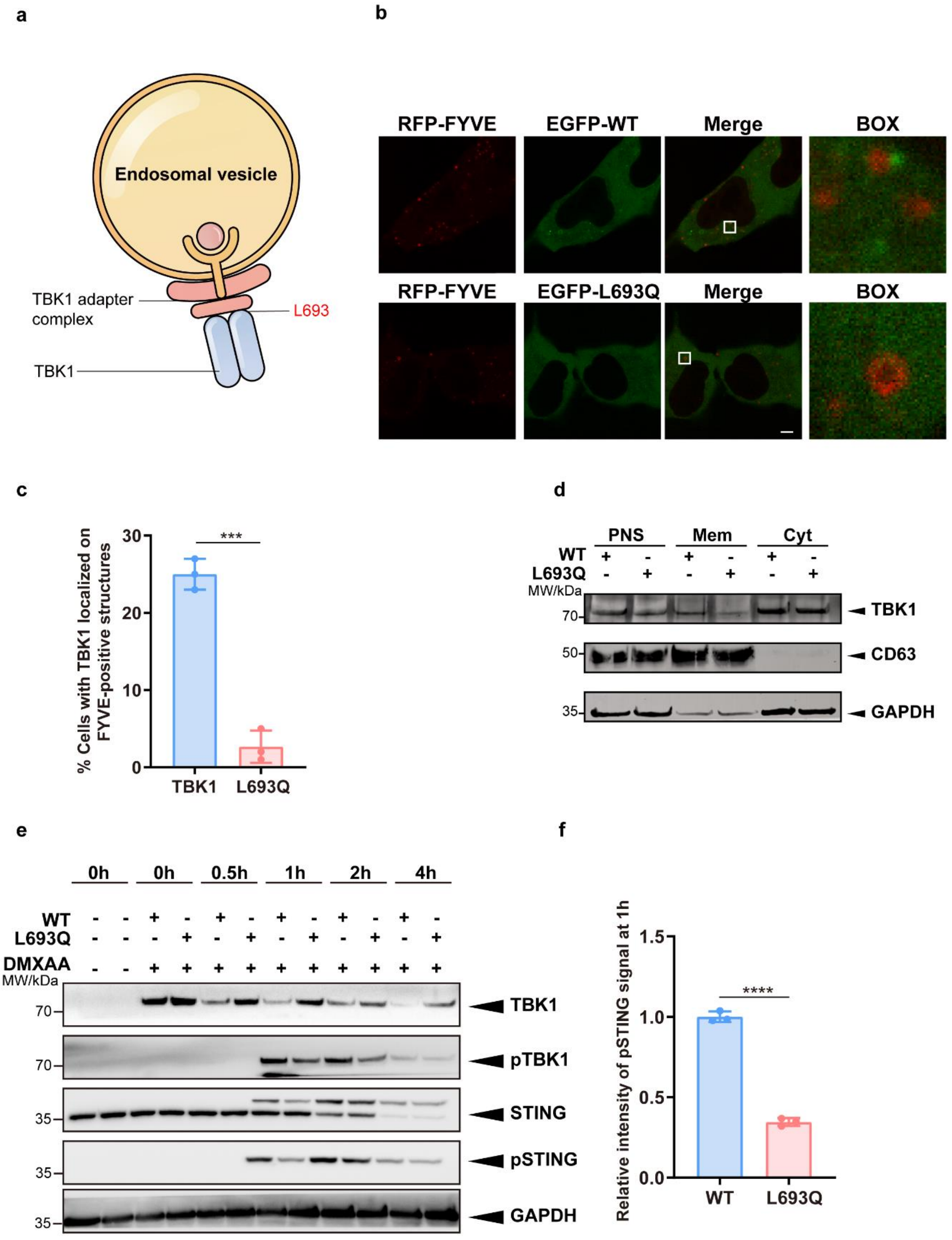

a
Endosomal vesicle
TBK1 adapter complex
L693
TBK1
b
RFP-FYVE
EGFP-WT
Merge
BOX
RFP-FYVE
EGFP-L693Q
Merge
BOX
c
% Cells with TBK1 localized on FYVE-positive structures
***
TBK1
L693Q
d
PNS
Mem
Cyt
WT
L693Q
MW/kDa
TBK1
CD63
GAPDH
e
0h
0h
0.5h
1h
2h
4h
WT
L693Q
DMXAA
MW/kDa
TBK1
pTBK1
STING
pSTING
GAPDH
f
Relative intensity of pSTING signal at 1h
****
WT
L693Q

Supplementary Figure S9 | Endosomal localization of TBK1 is critical for the STING signaling.

(a) A model showing that TBK1 is localized to endosomes by binding to adapter complexes.

(b) EGFP-TBK1 WT or L693Q was co-expressed with RFP-FYVE in TBK1/IKKε double knockout B16 cells. Protein localization was analyzed by confocal fluorescence microscopy. Scale bar = 5 μm.

(c) Percentage of cells with TBK1 localized on FYVE-positive structures, as shown in (b). 60 cells from each group were included in the count.

(d) Distribution of TBK1 WT and L693Q in membrane and cytosolic fractions. Cells were homogenized and then centrifuged at low speed to prepare post-nuclear supernatant (PNS). The PNS was then subjected to ultracentrifugation to separate membranes (Mem) from the cytosol (Cyt). Equal proportions of each fraction were loaded onto the gel. CD63 and GAPDH indicate membrane and cytosolic fractions, respectively.

(e) TBK1/IKKε double knockout B16 cells were rescued with TBK1 WT or L693Q. Cells were treated with 25 μg/mL DMXAA for various times, and the phosphorylation levels of TBK1 and STING at different time points were analyzed by immunoblotting.

(f) Statistical analysis of pSTING signal at 1h. The levels were determined by normalizing the gray values in (e).

One representative experiment of at least three independent experiments is shown. Data analyzed by two-tailed t-test and shown as mean ± SD ($n \geq 3$). ns, not significant, $p > 0.05$; * $p < 0.05$; ** $p < 0.01$; *** $p < 0.001$; **** $p < 0.0001$.

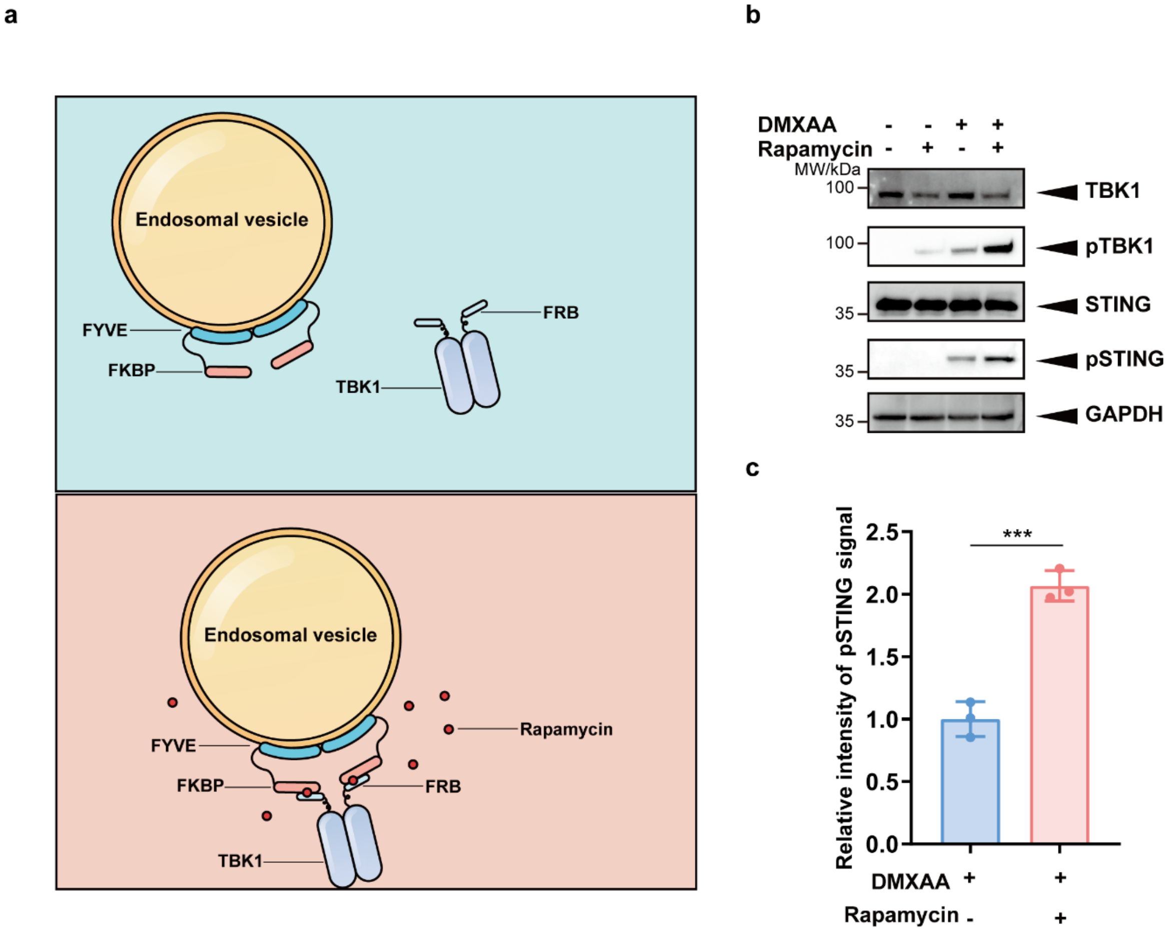

a
Endosomal vesicle
FYVE
FKBP
TBK1
FRB
Endosomal vesicle
FYVE
FKBP
TBK1
FRB
Rapamycin
b
DMXAA - - + +
Rapamycin - + - +
MW/kDa
100
100
35
35
35
TBK1
pTBK1
STING
pSTING
GAPDH
c
Relative intensity of pSTING signal
2.5
2.0
1.5
1.0
0.5
0.0
***
DMXAA + +
Rapamycin - +

Supplementary Figure S10 | Endosomal targeting of TBK1 enhances the STING signaling activation.

(a) A TBK1 endosome-targeting method based on the FKBP-FRB system. Vectors encoding endosome-targeting FYVE-FKBP and TBK1-FRB were co-expressed into TBK1/IKKε double knockout B16 cells. TBK1 was recruited to endosomes by rapamycin-induced FKBP-FRB binding.

(b) Cells in (a) were pretreated with rapamycin or DMSO for 1 h, respectively, followed by treatment with 25 μg/mL DMXAA for 1 h. The phosphorylation levels of TBK1 and STING at different time points were analyzed by immunoblotting.

(c) Statistical analysis of pSTING signal at 1h. The levels were determined by normalizing the gray values in (b).

One representative experiment of at least three independent experiments is shown. Data analyzed by two-tailed t-test and shown as mean ± SD (n ⩾ 3). ns, not significant, p > 0.05; * p < 0.05; ** p < 0.01; *** p < 0.001; **** p < 0.0001.

a

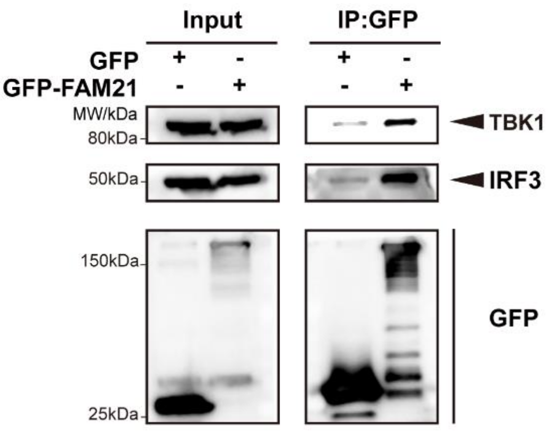

Input
IP:GFP
GFP
GFP-FAM21
MW/kDa
80kDa
50kDa
150kDa
25kDa
TBK1
IRF3
GFP

b

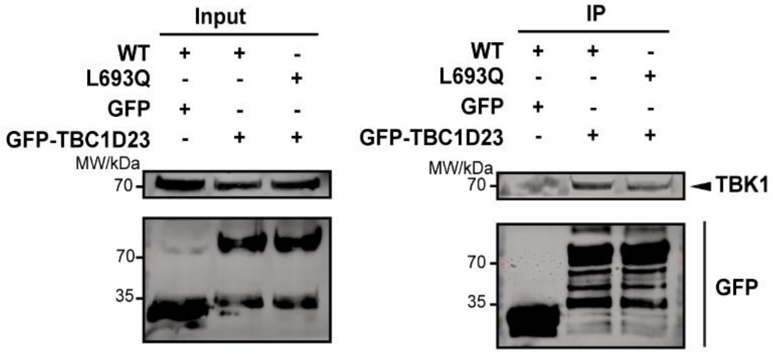

Input
IP
WT
L693Q
GFP
GFP-TBC1D23
MW/kDa
70
35
TBK1
GFP

c

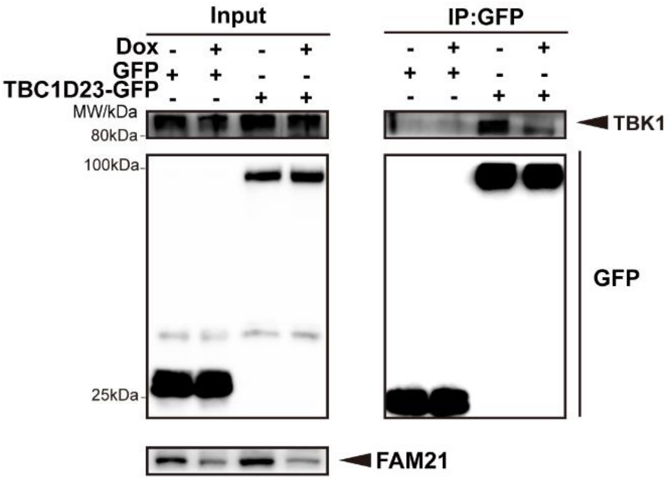

Input
IP:GFP
Dox
GFP
TBC1D23-GFP
MW/kDa
80kDa
100kDa
25kDa
TBK1
GFP
FAM21

Supplementary Figure S11 | FAM21 interacts with TBK1 and IRF3 and mediates the binding of TBC1D23 to TBK1.

(a) GFP-FAM21 was expressed in HEK293T cells and subjected to immunoprecipitation using GFP beads. Bound TBK1 and IRF3 were analyzed by immunoblotting.

(b) GFP or GFP-TBC1D23 was expressed in HEK293T cells and subjected to immunoprecipitation using GFP beads. TBK1/IKKε double knockout B16 cells were rescued with TBK1 WT or L693Q. Lysates were prepared, and incubated with GFP beads prebound with GFP or GFP-TBC1D23. Bound TBK1 was analyzed by immunoblotting.

(c) HEK293T cells stably expressing an inducible FAM21 knockdown system were treated with dox (1 μM) for 24 h. The cells were then were transiently expressed GFP or TBC1D23-GFP in the presence of dox for 48 hr, and subjected to immunoprecipitation using GFP beads. Bound TBK1 was analyzed by immunoblotting.

One representative experiment of at least three independent experiments is shown.

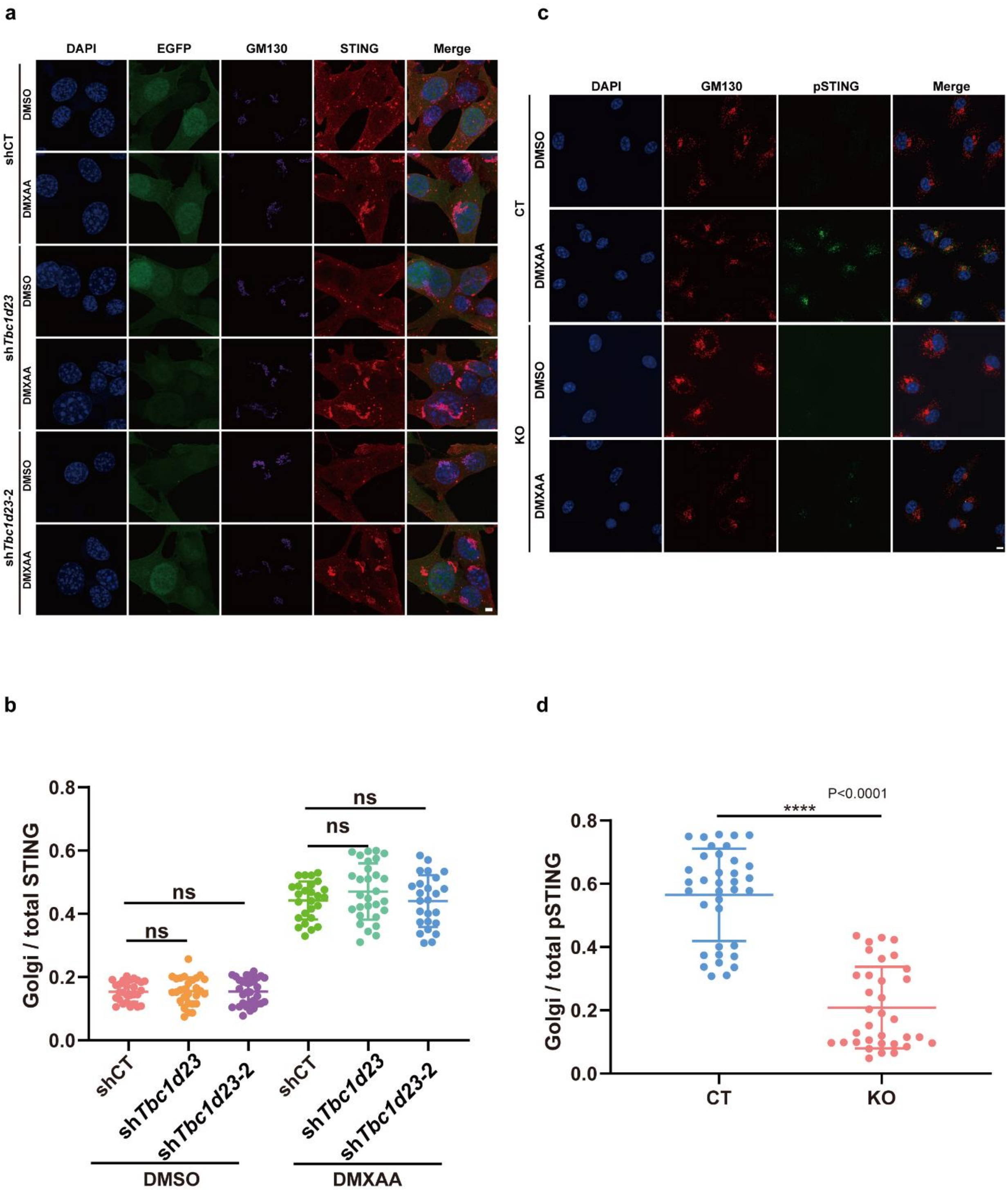
a
DAPI
EGFP
GM130
STING
Merge
shCT
DMSO
DMXAA
shTbc1d23
DMSO
DMXAA
shTbc1d23-2
DMSO
DMXAA
c
DAPI
GM130
pSTING
Merge
CT
DMSO
DMXAA
KO
DMSO
DMXAA
b
Golgi / total STING
0.8
0.6
0.4
0.2
0.0
ns
ns
ns
ns
shCT
shTbc1d23
shTbc1d23-2
shCT
shTbc1d23
shTbc1d23-2
DMSO
DMXAA
d
P<0.0001
****
Golgi / total pSTING
0.8
0.6
0.4
0.2
0.0
CT
KO

Supplementary Figure S12 | TBC1D23 is indispensable for STING phosphorylation, but not for STING translocation to Golgi.

(a) MEFs knock down TBC1D23 by stably expressed shRNA (shTbc1d23, shTbc1d23-2), cells expressing untargeted shRNA served as a control (shCT). Cells were treated with DMXAA (25 μg/mL) for 30 min. Golgi-association of STING was analyzed by confocal fluorescence microscopy, using Golgi-resident protein GM130 as a reference. Scale bar = 5 μm.

(b) BMDM cells were treated with DMXAA (25 μg/mL) for 30 min. Golgi-association of pSTING was analyzed by confocal fluorescence microscopy, using Golgi-resident protein GM130 as a reference. Scale bar = 5 μm.

(c-d) Quantitative analysis (a-b) of the ratio of Golgi fluorescence intensity to total fluorescence intensity.

One representative experiment of at least three independent experiments is shown. Data analyzed by two-tailed t-test and shown as mean ± SD ($n \geqslant 3$). ns, not significant, $p > 0.05$; * $p < 0.05$; ** $p < 0.01$; *** $p < 0.001$; **** $p < 0.0001$.

Supplementary Table S1. Sequence of qPCR primers used in this study.

Target	Forward (5’ - 3’)	Reverse (5’ - 3’)
GAPDH	AGCCACATCGCTCAGACAC	GCCCAATACGACCAAATCC
IFNB1	GATTCATCTAGCACTGGCTGG	CTTCAGGTAATGCAGAATCC
TNF-α	TGTAGCCCATGTTGTAGCAAACC	GAGGACCTGGGAGTAGATGAGGTA
Rpl13a	AGTATCTGGCCTTTCTCCGG	CCGAACAACCTTGAGAGCAG
Ifnb1	AGCTCCAAGAAAGGACGAACAT	GCCCTGTAGGTGAGGTTGATCT
Tnf-α	CATCTTCTCAAAATTCGAGTGACAA	CCAGCTGCTCCTCCACTTG
Il-6	TGAACAACGATGATGCACTTGC	GCTATGGTACTCCAGAAGACC
Tbc1d23	GATAGTTTGGCATCATGGGATGG	CCAGGAGTAATTCTGCTGCCT

Supplementary Table S2. Sequence of gene knock down and knock out target used in this study

Target gene	sequence
Tbc1d23	CAGGAGGTTGTGATCTTGAAA
Tbc1d23-2	TGAGCAGAACACTATTCATAA
GOLGA1	GAAGAGACTAGAACAGAACTT
GOLGA4	CGTGATGCAAAGAACTTAATT
FAM91A1	GCTCAGATGTAAATGGGAGTA
TBC1D23	GCATGTTACTGTTCCACTGAA
WDR11	GCAGTCGTATTCAGAGATAAA
FAM21	GCTGTGAACTATGGCTTACAA
Tbk1 gRNA	AGACAGGAGCCACAGATGGT
Ikkε gRNA	GGGAGTTTGAGGTCCTGCGG
